# Mechanism of *Paeoniae Radix Alba* in the Treatment of Non-alcoholic Fatty Liver Disease Based on Sequential Metabolites Identification Approach, Network Pharmacology, and Binding Affinity Measurement

**DOI:** 10.3389/fnut.2021.677659

**Published:** 2021-09-16

**Authors:** Zhiqiang Luo, Yang Liu, Xing Han, Wenning Yang, Guopeng Wang, Jing Wang, Xiaoquan Jiang, Muli Sen, Xueyan Li, Guohua Yu, Yuanyuan Shi

**Affiliations:** ^1^School of Life Sciences, Beijing University of Chinese Medicine, Beijing, China; ^2^School of Chinese Materia Medica, Beijing University of Chinese Medicine, Beijing, China; ^3^State Key Laboratory of Natural and Biomimetic Drugs, School of Pharmaceutical Sciences, Peking University, Beijing, China; ^4^Zhongcai Health (Beijing) Biological Technology Development Co., Ltd., Beijing, China; ^5^Department of Biomedical Engineering, Shenzhen Research Institute, Beijing University of Chinese Medicine, Shenzhen, China

**Keywords:** *Paeoniae Radix Alba*, non-alcoholic fatty liver disease, sequential metabolism, UPLC-Q Exactive Orbitrap HRMS, surface plasmon resonance, network pharmacology

## Abstract

Screening functional food ingredients (FFI) from medicinal and edible plants (MEP) has still remained a great challenge due to the complexity of MEP and its obscure function mechanisms. Herein, an integrated strategy based on sequential metabolites identification approach, network pharmacology, molecular docking, and surface plasmon resonance (SPR) analysis was proposed for quickly identifying the active constituents in MEP. First, the sequential biotransformation process of MEP, including intestinal absorption and metabolism, and hepatic metabolism, was investigated by oral gavage, and intestinal perfusion with venous sampling method. Then the blood samples were analyzed by UPLC-Q Exactive Orbitrap HRMS. Second, the network pharmacology approach was used to explore the potential targets and possible mechanisms of the *in vivo* metabolites of MEP. Third, molecular docking and SPR approaches were used to verify the specific interactions between protein targets and representative ingredients. The proposed integrated strategy was successfully used to explore the heptoprotective components and the underlying molecular mechanism of *Paeoniae Radix Alba* (PRA). A total of 44 compounds were identified in blood samples, including 17 porotypes and 27 metabolites. The associated metabolic pathways were oxidation, methylation, sulfation, and glucuronidation. After further screening, 31 bioactive candidates and 377 related targets were obtained. In addition, the bioactive components contained in PRA may have therapeutic potentials for non-alcoholic fatty liver disease (NAFLD). The above results demonstrated the proposed strategy may provide a feasible tool for screening FFI and elaborating the complex function mechanisms of MEP.

## Introduction

Medicinal and edible plants (MEP) has been extensively used for preventing and treating various diseases in China for centuries (1). After taken orally, MEP would be bio-transformed progressively and orderly from the gastrointestinal tract and liver, to the systemic blood stream (2, 3). However, current metabolic studies are just focus on characterizing the metabolites in different biological samples, like urine, blood, bile and other tissues/organs, and they could not comprehensively illustrate the dynamic biotransformation process of MEP (4). In addition, accumulating evidences have suggested that gut wall metabolism plays an important role in first pass metabolism of small molecules, while only a few *in vitro* studies using intestinal microsomes have been performed (5). Recently, many *in situ* approaches have been developed and demonstrated efficient to study drug intestinal absorption and metabolism, such as *in situ* closed-loop method (4), intestinal single-pass perfusion, intestinal recirculating perfusion, and intestinal perfusion with venous sampling (IPVS) (6). Among them, IPVS is recommended as it enables intestinal absorption/metabolism to occur at body temperature, and allows gut wall metabolism to be studied without interference by the confounding effects of liver metabolism (7, 8). Moreover, detecting and identifying the absorbed components and metabolites of MEP from complex plasma samples is often challenging, due to the extremely low concentrations of the interested compounds and the interference of the endogenous metabolites and proteins (9). Owing to the high sensitivity and selectivity, ultrahigh performance liquid chromatography coupled with hybrid Q-Exactive-Orbitrap high resolution mass spectrometry (UPLC-Q Exactive Orbitrap HRMS) has become a powerful tool for rapidly and accurately profiling the trace compounds in biological samples (10). Therefore, screening the prototypes and metabolites involved in the dynamic metabolic process using UPLC-Q Exactive Orbitrap HRMS and IPVS is the first step to systemically explicate material basis of MEP.

Unlike western medicine of “one target, one drug,” MEP is a complicated system with multi-component and multi-target characteristics, which achieves its therapeutic effects through targeting multiple physiological pathways (11). Due to the complicated chemical components in MEP, conventional approaches have great difficulties in delivering a systematic understanding of the synergistic effects of MEP for preventing and treating complex diseases (12). With bioinformatics' rapid progress, the newly emerging network pharmacology has greatly facilitated mechanistic studies into the synergistic actions of multi-component drugs at the proteome or systemic level (13). It emphasizes the concept of “network target, multi-component therapeutics,” which is consistent with the integrality and systematicness of traditional Chinese medicine (TCM) theory (14, 15). Up to date, this state-of-the-art method has been successfully used for elucidating the complex molecular mechanisms of TCM for the treatment of various diseases, such as Alzheimer's disease (2), cardiovascular disease (14, 16), cancer (17, 18), diabetes (19), asthma (20, 21), gastritis (22), acute ulcerative colitis (23), and acute mountain sickness (24).

Surface plasmon resonance (SPR) biosensor is a powerful tool for characterizing and quantifying the kinetics and binding affinities of biomolecular interactions (25, 26). When the analyte molecules in a liquid sample were in contact with the ligands, changes in the refractive index (RI) at the sensor surface were produced, which can be measured by the optical reader (27, 28). Hence, the major advantage of SPR is that the sample can be detected in real time and without the need of labeling (29, 30). Another advantage is that SPR can directly and specifically capture the bioactive candidates from complex matrices (31). Therefore, SPR has been increasingly penetrated into almost all fields of TCM research, such as bioactive compound screening and target fishing (32).

*Paeoniae Radix Alba* (PRA, Baishao in Chinese), the dried roots of *Paeonia lactiflora* Pall., is a famous herbal medicine or functional food in China and many other Asia countries (33). According to TCM theory, PRA has antispasmodic, tonic, astringent, and analgesic properties (34), which has been clinically used as an anticoagulant, antidepressant, antioxidant, and liver protectant agent for centuries (33). Recently, accumulating evidences indicate that PRA exerts significant effects in the prevention and treatment of liver diseases, such as acute liver injury (35), fatty liver diseases (36, 37), liver fibrosis (38, 39), cholestasis (40, 41), hepatitis (42), and liver cancer (43, 44). However, the bioactive components and the underlying molecular mechanisms are largely unknown.

**Figure 1:**
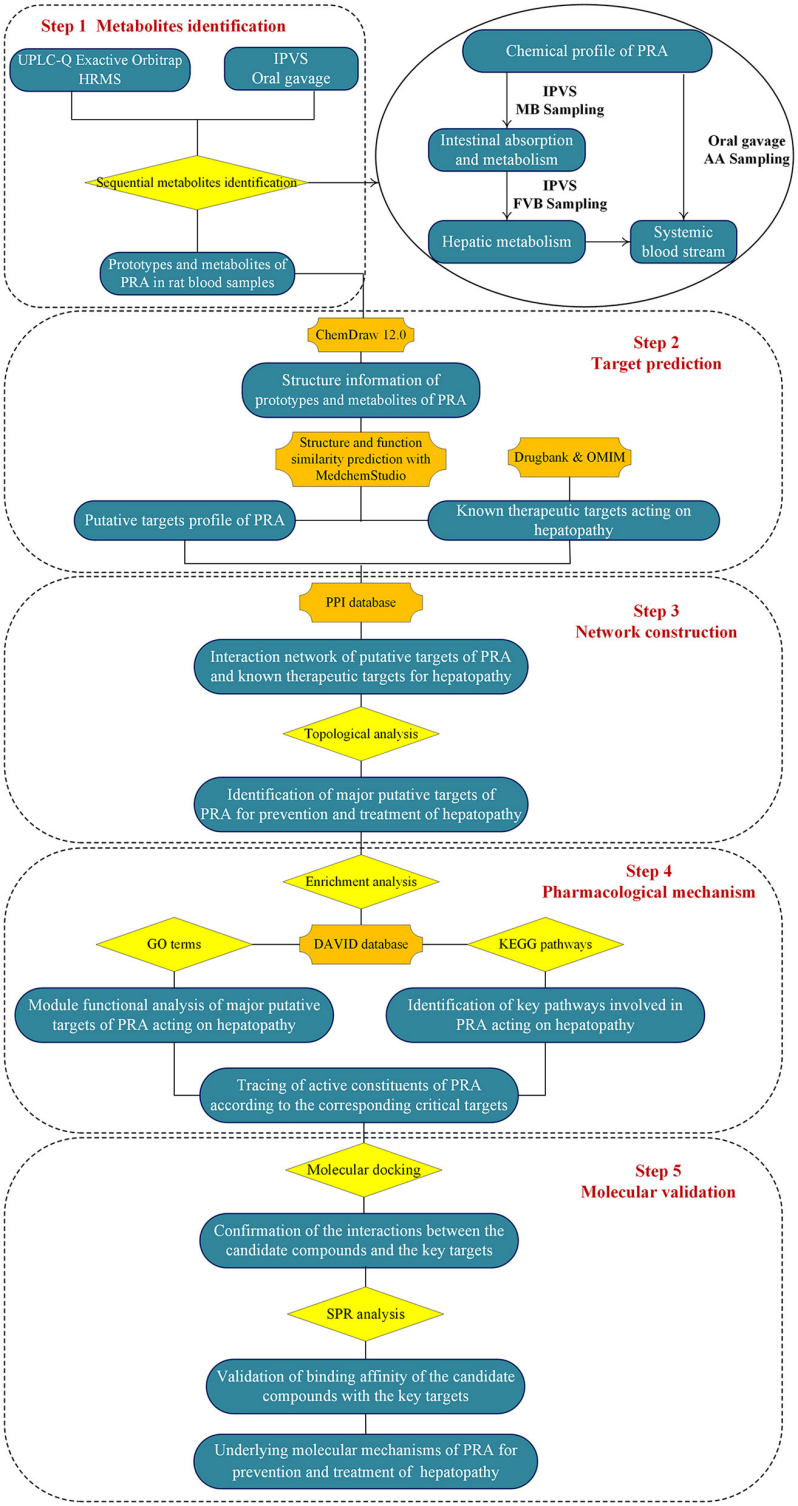
Flowchart of the study design.

To address the above issues, an integrated strategy was developed for quickly identifying functional ingredients from MEP, using PRA as an example. First, the chemical profile of MEP extract was identified by UPLC-Q Exactive Orbitrap HRMS. Second, the intestinal absorption and metabolism of MEP was investigated by intestinal perfusion with mesenteric blood (MB) sampling. By comparing the components in mesenteric blood with the chemical components of MEP, we could identify the absorbed components and metabolites produced by gut wall metabolism. Third, the hepatic metabolism was investigated by intestinal perfusion with femoral venous blood (FVB) sampling. By comparing the components in mesenteric blood with the components in femoral venous blood, we could identify the components in the systemic blood stream and metabolites produced by hepatic metabolism. Fourth, to comprehensively screen the components *in vivo*, the major components and metabolites in rat plasma [blood was collected from the abdominal aorta (AA)] after oral administration of MEP extract were analyzed. Fifth, the network pharmacology strategy was used to screen the molecular targets and pathways involved in MEP for preventing and treating particular diseases. Finally, molecular docking and surface plasmon resonance methodologies were employed to confirm the binding abilities between the candidate compounds and their associated targets. We hope this integrated strategy would be helpful to identify the functional food ingredients (FFI) from MEP. The flowchart of the study design was illustrated in Figure 1.

## Materials and Methods

### Materials and Reagents

*Paeoniae Radix Alba* was supplied by Lanzhou Foci Pharmaceutical Co., Ltd. (Qinghai, China) and authenticated by Professor Jingjuan Wang (Beijing University of Chinese Medicine, Beijing, China). Chromatographic-grade acetonitrile, menthol and formic acid was supplied by Fisher Scientific. Reference standards (purity ≥ 90%) of paeoniflorin, gallic acid, benzoic acid were purchased from National Institutes for Food and Drug Control (Beijing, China). Reference standards (purity ≥ 90%) of benzoylpaeoniflorin, oxypaeoniflorin, albiflorin, and 1,2,3,4,6-O-pentagalloylglucose were purchased from Shanghai Yuanye Biological Technology Co., Ltd. (Shanghai, China). Ultrapure water was purified by the Millipore Milli Q plus purification system. All other reagents used were of analytical grade and commercially available.

### Preparation of PRA Solution

PRA was chopped into pieces, and then PRA pieces (about 300 g) were decocted with water twice (solid-liquid ratio: 1:10), 1 h for each time. The supernatant was then filtered and the two filtrates were mixed and condensed to obtain a PRA solution (1 g/mL) which was used for animal studies. For chemical analysis, the PRA solution (1 g/mL) was diluted to 10 mg/ml crude drug and then filtered with 0.22 μm membrane before UPLC-Q Exactive Orbitrap HRMS.

### Preparation of Standard Solutions

Individual stock solutions of 7 reference standards were dissolved with methanol in a 10 mL volumetric flask and stored at 4°C. Then the standard solutions were filtered with 0.22 μm membrane before UPLC-Q Exactive Orbitrap HRMS.

### Animals

Pathogen free male Sprague-Dawley rats (280–300 g) were purchased from Spfanimals Laboratory Animal Technology Co. Ltd (Beijing, China). Treatment for animals in this study was approved by the animal ethnic committee of Beijing University of Chinese Medicine. The rats had free access to water and standard diet, and were maintained in an environmentally controlled rearing room (temperature:23°C, the humidity: 60%) under a 12 h light/dark cycle. All animals were acclimated for at least a week, then fasted for ~12 h with water *ad libitum* before each experiment.

### Surgical Procedures of IPVS

For animals undergoing IPVS experiments, the surgical procedures were conducted followed previously published reports (45). Briefly, prior to initiation of perfusion surgical operation, several rats were used for donor blood. Whole blood was drawn from the abdominal aorta using a 10-mL syringe (100 U of heparin added to 10 mL of donor blood) and incubated in a 37°C water bath until administered to the recipient rat. The recipient rat was anesthetized by intraperitoneal injection of chloral hydrate (400 mg/kg), fixed in a supine on the operating table, and kept warm by a heat lamp placed over the surgical area.

Upon verification of the loss of pain reflex, the left external jugular vein was exposed and cannulated with a 24-gauge i.v. catheter to transfuse blood from the donor blood reservoir. Then the abdominal cavity was opened along the abdominal line. The jejunum segment was located and the two ends were incised with surgical scissors for cannula. Two silicone tubes were inserted through the small slits and secured. The segment was then rinsed with warm isotonic saline until the effluent was clear. To collect venous outflow, a 24-gauge i.v. catheter filled with heparinized saline was intubated into the mesenteric vein (gut wall metabolism)/femoral vein (hepatic metabolism) and secured with instant glue. PRA solution (1 g/mL) was incubated in a 37°C water bath to maintain the temperature and pumped at a flow rate of 0.2 mL/min. The blood was pumped at the flow rate of 0.3 mL/min. At the end of the surgical procedure and throughout the experiment, the exposed intestinal segment was covered with a piece of sterilized gauze that had been moistened by frequent applications of warm isotonic saline.

Blood draining from the cannulated mesenteric vein was collected into heparinized centrifuge tubes within 2 h. Plasma samples were separated by centrifuging the blood samples at 4,000 rpm for 10 min and stored at −20°C.

### Oral Drug Administration

Rats were randomly divided into eight groups (three animals each). Then, the four treatment group rats were administrated by oral gavage of 4 mL PRA solution (1 g/mL). The corresponding blank groups were given 4 mL saline instead. The rats were anesthetized by intraperitoneal injection of chloral hydrate (400 mg/kg). Then, blood samples were collected from the abdominal aorta at 0.5, 1, 1.5, and 2 h (three rats for each time), respectively. At the end of this study, all rats were sacrificed by conducting a bilateral thoracotomy.

### Preparation of the Blood Samples

An aliquot of 1.5 mL of plasma sample was added into 1.5 mL of 4% phosphoric acid. Then the mixture was purified by solid phase extraction (SPE) on an Oasis PRiME HLB cartridge. The sample was loaded on the pretreated column, washed with 3 mL of water, and then eluted with 6 mL of acetonitrile: methanol (90:10 v/v). The eluate was collected and dried by nitrogen blowing instrument. The residue was dissolved in 1.5 mL of methanol and filtered with 0.22 μm membrane before LC/MS analysis.

### LC/MS Analysis

For the LC/MS analysis, an Q Exactive Orbitrap high resolution mass spectrometer equipped with a heated electrospray ionization (HESI) source, was coupled to a Thermo Dionex Ultimate 3000 UPLC system (consisting of an autosampler, a diode array detector, a column oven and a dual pump connected to an online degasser). The data were recorded and processed using Xcalibur, Metworks and Mass Frontier 6.0 software packages (Thermo Fisher Scientific).

UPLC chromatographic separations were executed on a Waters CORTECS UPLC T3 (2.1 × 100 mm, 1.6 μm) column thermostated at 40°C. The mobile phases consisted of water with 0.1% formic acid (A) and acetonitrile (B), and the gradient program was conducted as follows: 0–1 min (5% B), 1–20 min (5–95% B), 20–21 min (95–95% B), 21–21.1 min (95–5% B), and 21.1–22 min (5–5% B). The sample flow rate was 0.3 mL/min and the injection volume was 2 μL.

The MS conditions were as follows: alternate switching (−)/(+) ESI full scan mode, the capillary temperature was 300°C, auxiliary temperature was 250°C, positive spray voltage was set at +3.5 kV, negative spray voltage was set at −3.0 kV, shealth gas (N_2_) flow was 35 Arb, aux gas flow rate was 10 Arb. Full MS scans were acquired in the range of *m/z* 100–1,500, the collision energy was set at 20, 30, 40 eV. The MS/MS experiments were set as data-dependent scans.

### Predicting the PRA-Related Targets

As described in our previous studies (14, 22), a powerful drug similarity search tool named MedChem Studio (MedChem Studio, 3.0; Simulations Plus, Inc, Lancaster, CA, USA, 2012) was employed for the prediction of the PRA-related targets, with a similarity threshold of 0.60.

### Collection of Hepatopathy (HP)-Related Targets

By using the query of “liver disease” and “hepatopathy,” and limiting the species with “*Homo sapiens*,” the HP-related targets were searched from DrugBank database (https://go.drugbank.com/, version 5.1.7, updated on July 2nd, 2020), and the Online Mendelian Inheritance in Man (OMIM) database (https://omim.org/, updated on July 1st, 2019). DrugBank is a public database which integrates the drug structures (including approved, investigational and withdrawn drugs), drug target proteins/genes, pathways and other information associated with human diseases (46). OMIM can provide detailed information on genetic disorders and their related human genes (47). To reduce the false positives, only the druggable proteins/genes links to the HP were retained.

### Protein-Protein Interaction (PPI) Data

The PRA- and HP-related targets were input into STRING (Search Tool for the Retrieval of Interacting Genes/Proteins) database (http://string-db.org/, version 11.0) to predict the possible PPI information. The STRING database is a versatile platform for the straightforward identification of direct or indirect functional interactions between proteins (48). Then, the PPI data whose confidence scores >0.4 would be reserved.

### Network Construction and Analysis

To elaborate the connections among the components, target proteins/genes, and disease, a “component-target-disease” network was created by inputting the data of selected components of PRA, PRA-associated targets as well as the targets related to HP into Cytoscape software (version 3.6.0, Boston, MA, USA). Cytoscape is a valuable platform for the analysis and visualization of the complicated network involved in various biological processes (49). Then, node degree, a quantitative characteristic of the network, was calculated. Nodes with the degree values higher than twice the median degree of the whole nodes would be chosen as a hub (14). Next, the interaction network of hubs was established by using the direct links between hubs. Furthermore, the network analyzer function was used for the analysis of degree centrality (DC), betweenness centrality (BC), and closeness centrality (CC) to assess the topological importance of a node in the entire network, as described in our prior publications (22). To increase the reliability of the predicted results, the hubs with “degree” > median DC, “betweenness” > median BC and “closeness” > median CC were recognized as critical hubs.

### Pathway Enrichment Performance

By using DAVID (Database for Annotation, Visualization and Integrated Discovery) bioinformatics resources (http://david.abcc.ncifcrf.gov/home.jsp/, version 6.7), Kyoto Encyclopedia of Genes and Genomes (KEGG) pathway (KEGG, http://www.genome.jp/kegg/) and gene ontology (GO) enrichment evaluation were performed to clarify the pathways that were involved in PRA acting on liver disease. A *P* < 0.05 was considered high confidence.

### Molecular Docking Simulation

To further confirm the interactions between the candidate compounds and the key targets, molecular docking analysis was performed by use of the CDOCKER module implemented in Discovery Studio 2016 (DS 2016). The crystallographic structures of the target proteins were retrieved from the protein data bank (PDB, http://www.rcsb.org/pdb/home/home.do), and prepared by adding hydrogens, deleting the ligands and water motifs. The three-dimensional (3D) structures of the potential active components were drawn with Chem3D Pro 12.0. CDOCKER interaction energies (CIEs) were adopt to evaluate the binding abilities between the vital target proteins and their corresponding constituents.

### SPR Analysis

According to the molecular docking results, PRKAG1 and NFKB1 were chosen for SPR analysis. A Biacore 8K (GE Healthcare, Sweden) was used to perform the SPR experiments. A freshly prepared mixture of NHS and EDC (1:1, v/v) was first injected into the instrument to activate the carboxyl groups on the surface of the CM5 sensor (General Electric Company, USA). Then, proteins were diluted in sodium acetate solution (GE Healthcare) and immobilized on CM5 chips by using the Amine Coupling Kit (GE Healthcare), with immobilization levels of 2200 and 11800 RU (response units), respectively. The affinity measurement was carried out following the protocol provided by GE Healthcare. To exclude false-positive results, a reference channel without the conjugated protein was activated and blocked for each analysis, which served as a control to test for unspecific binding to the chip. Analytes were serially injected at a flow rate of 30 μL/min. The spontaneous association-dissociation process of the component on the protein was real-time monitored by the response value. The SPR curves were finally fitted by use of Biacore Insight Evaluation Software according to 1:1 Langmuir binding model, from which the binding constants and kinetic parameters were calculated.

## Results and Discussion

### Sequential Metabolism of PRA

**Figure 2:**
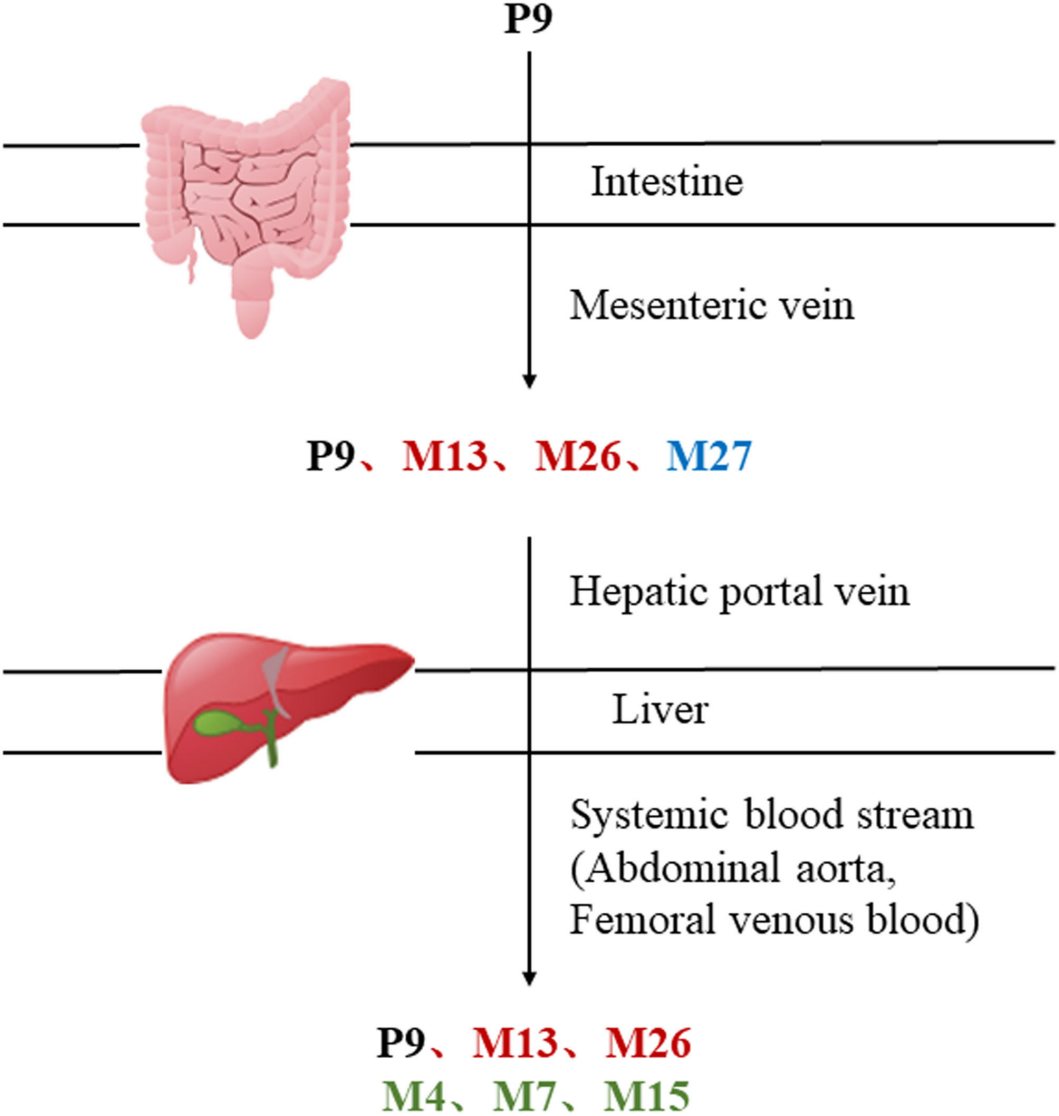
The sequential process of paeoniflorin in the digestive system.

MEP taken by oral administration pass sequentially from the gastrointestinal lumen, through the gut wall and liver, and then reach the systemic circulation (50), which are subject to extensive “first-pass” elimination in many cases (51). In this work, the detection and identification of components in blank plasma sample, drug-treated plasma sample and PRA sample were conducted by UPLC-Q Exactive Orbitrap HRMS. As depicted in Supplementary Figures 1, 2 and Table 1, a total of 40 components were first identified from the water extract of PRA, mainly including monoterpenes and their glycosides, phenolic compounds, and tannins. We selected oxypaeoniflorin (peak 19), gallic acid (peak 3) and 1,2,3,4,6-penta-O-galloyl-β-D-glucose (peak 29) as the representative components to illustrate the characteristic fragmentation rules of monoterpenes, phenolic compounds and tannins, respectively (Supplementary Figures 3–5). Further, 44 compounds (Table 2), including 17 prototypes and 27 metabolites were identified from drug-treated plasma sample through comparing their molecular formulas, fragment ions, and retention times with those of the parent compounds. Among them, 32 of them were from MB group, 21 were from FVB group, and 29 were from AA group. The main metabolic pathways of PRA were found, including oxidation, methylation, sulfation, glucuronidation (Supplementary Figures 6–12). It should be noted that 15 metabolites were produced after passing the intestine, which may be mainly due to the metabolizing enzymes present in gut wall, suggesting that the intestine may play an important role in the first-pass metabolism of MEP. In addition, most metabolites of PRA were generated after hepatic metabolism, indicating that liver was the major metabolic site of PRA. The sequential metabolism of PRA was clearly characterized and paeoniflorin (P9) was selected as an example, as shown in Figure 2. Taken together, the above study could give a comprehensive map of the dynamic metabolic process of PRA, which would effectively narrow the range of potentially bioactive components of PRA.

**Table 1:** Identification of components in PRA by UPLC-Q Exactive Orbitrap HRMS.

**Peak No**.	***t_***R***_* (min)**	**Measured Mass**	**Error (ppm)**	**Molecular formula**	**MS/MS fragments**	**Identification compound**
1	1.27	421.13156 [M+HCOO]^−^	2.630	C_16_H_24_O_10_	345.12, 195.07, 183.07, 151.08	Desbenzoylpaeoniflorin or isomer
2	1.43	421.13156 [M+HCOO]^−^	2.630	C_16_H_24_O_10_	345.12, 195.07, 183.07, 151.08	Desbenzoylpaeoniflorin or isomer
3	1.60	169.01332 [M-H]^−^	1.007	C_7_H_6_O_5_	125.02	Gallic acid*
4	1.64	125.02325 [M-H]^−^	−0.564	C_6_H_6_O_3_	97.03	Pyrogallol
5	1.70	405.14017 [M+HCOO]^−^	2.547	C_16_H_24_O_9_	314.85, 197.08, 153.09, 135.08	1-O-glucopyranosyl paeonisuffrone
6	1.94	493.11987 [M-H]^−^	2.177	C_19_H_26_O_15_	313.06, 283.05, 169.01, 125.02	1′-O-galloylsucrose or isomer
7	2.11	493.11954 [M-H]^−^	1.508	C_19_H_26_O_15_	313.06, 271.05, 169.01	1′-O-galloylsucrose or isomer
8	2.14	493.11990 [M-H]^−^	2.238	C_19_H_26_O_15_	313.06, 283.05, 169.01, 125.02	4′-O-galloylsucrose or isomer
9	2.17	493.11954 [M-H]^−^	1.508	C_19_H_26_O_15_	313.06, 271.05, 211.02, 169.01	Diglucosyl gallic acid
10	2.20	493.11981 [M-H]^−^	2.056	C_19_H_26_O_15_	313.06, 283.05, 169.01, 125.02	6′-O-galloylsucrose or isomer
11	2.34	331.06708 [M-H]^−^	3.344	C_13_H_16_O_10_	193.01, 169.01, 151.00, 125.02	6-O-galloyl-β-D-glucopyraneose
12	3.39	331.06567 [M-H]^−^	−0.915	C_13_H_16_O_10_	168.01, 125.02	Glucogallin
13	3.42	527.14038 [M-H]^−^	1.609	C_23_H_28_O_14_	313.06, 169.01, 151.00, 165.05, 125.02	6′-O-galloyl desbenzoylpaeoniflorin
14	3.99	389.14542 [M+HCOO]^−^	3.075	C_16_H_24_O_8_	181.09, 163.08, 113.02	Moudanpioside F
15	4.04	137.02332 [M-H]^−^	−0.004	C_7_H_6_O_3_	119.01,93.03	3,4-Dihydroxybenzaldehyde
16	4.52	495.1507 [M-H]^−^	2.014	C_23_H_28_O_12_	345.12, 281.07, 137.02, 93.03	ortho-Oxypaeoniflorin
17	4.65	361.15045 [M-H]^−^	3.160	C_16_H_26_O_9_	161.04, 113.02, 101.02	6-O-glucopyranosyl-lactinolide
18	4.69	289.0715 [M-H]^−^	2.89	C_15_H_14_O_6_	245.08, 203.07, 151.04, 125.02, 109.03	(+)-Catechin
19	4.76	495.1507 [M-H]^−^	2.014	C_23_H_28_O_12_	465.14, 333.10, 165.05, 137.02, 93.03	Oxypaeoniflorin*
20	5.32	543.11768 [M-H]^−^	1.827	C_23_H_28_O_13_S	259.03, 213.02, 121.03	Paeoniflorin sulfite
21	5.55	643.2223 [M+H]^+^	−2.363	C_29_H_38_O_16_	197.08, 151.08, 105.03	Glucopyranosylalbiorin
22	5.60	687.21423 [M+HCOO]^−^	1.658	C_29_H_38_O_16_	165.05, 121.03, 101.02	Isomaltopaeoniflorin or isomer
23	5.72	319.11719 [M+H]^+^	−3.05	C_17_H_18_O_6_	301.11, 197.08, 179.07, 151.08, 105.03	Paeoniflorigenone
24	5.74	525.16095 [M+HCOO]^−^	1.300	C_23_H_28_O_11_	479.15, 167.03, 121.03	Albiflorin*
25	5.80	687.21399 [M+HCOO]^−^	1.309	C_29_H_38_O_16_	165.05, 121.03, 101.02	Isomaltopaeoniflorin or isomer
26	5.90	525.16089 [M+HCOO]^−^	1.186	C_23_H_28_O_11_	479.15, 167.03, 121.03	Isopaeoniflorin
27	6.00	525.16095 [M+HCOO]^−^	1.300	C_23_H_28_O_11_	479.15, 167.03, 121.03	Paeoniflorin*
28	6.85	631.16675 [M-H]^−^	1.59	C_30_H_32_O_15_	465.14, 313.06, 271.05, 211.02, 169.01, 151.00, 121.03	4-O-galloylalbiflorin
29	6.88	939.11182 [M+HCOO]^−^	2.143	C_41_H_32_O_26_	769.09, 617.08, 465.07, 313.06, 295.05 169.01, 125.02	1,2,3,4,6-penta-O-galloyl-β-D-glucose*
30	7.06	121.02834 [M-H]^−^	−0.545	C_7_H_6_O_2_	121.03	Benzoic acid*
31	7.30	631.16675 [M-H]^−^	1.59	C_30_H_32_O_15_	465.14, 313.06, 271.05, 211.02, 169.01, 151.00, 121.03	6′-O-galloylalbiflorin
32	7.61	525.16101 [M-H]^−^	1.414	C_24_H_30_O_13_	195.07, 121.03	Mudanpioside E
33	7.64	509.16611 [M-H]^−^	1.487	C_24_H_30_O_12_	121.03, 101.02	Mudanpioside D
34	7.68	509.16617 [M+HCOO]^−^	1.605	C_23_H_28_O_10_	121.03, 101.02	1-O-β-D-glucopyranosyl-8-O-benzoylpaeonisuffrone
35	7.93	987.3129 [M+HCOO]^−^	1.981	C_46_H_54_O_21_	327.11, 177.05, 165.05, 121.03	Paeonidanin E
36	8.16	599.17725 [M-H]^−^	2.224	C_30_H_32_O_13_	281.07, 137.02, 121.03	Benzoyloxypaeoniflorin
37	8.17	615.17206 [M-H]^−^	1.996	C_30_H_32_O_14_	493.14, 313.06, 169.01, 151.00, 121.03	Moudanpioside H
38	9.18	629.18744 [M-H]^−^	1.523	C_31_H_34_O_14_	311.08, 167.03, 121.03	Mudanpioside B
39	9.47	629.18790 [M+HCOO]^−^	1.523	C_30_H_32_O_12_	165.05, 121.03	Benzoylpaeoniflorin*
40	9.64	629.18790 [M+HCOO]^−^	1.618	C_30_H_32_O_12_	165.05, 121.03	Benzoylalbiflorin

t_R_, retention time; *Compared with reference standards.

**Table 2:** Identification of prototypes and metabolites of PRA in different plasma samples by UPLC-Q Exactive Orbitrap HRMS.

**Peak No**.	***t_***R***_* (min)**	**Measured mass**	**Molecular formula**	**Error (ppm)**	**Prototypes/Parent compounds**	**Metabolites**	**MB**	**FVB**	**AA**
P1	4.04	137.02332 [M-H]^−^	C_7_H_6_O_3_	−0.004	3,4-Dihydroxybenzaldehyde	–	+	+	+
P2	4.52	495.15070 [M-H]^−^	C_23_H_28_O_12_	2.014	ortho-Oxypaeoniflorin	–	+	–	–
P3	4.78	495.15070 [M-H]^−^	C_23_H_28_O_12_	2.014	Oxypaeoniflorin	-	+	–	–
P4	5.32	543.11768 [M–H]^−^	C_23_H_28_O_13_S	1.827	Paeoniflorin sulfite	–	+	+	–
P5	5.60	687.21423 [M+HCOO]^−^	C_29_H_38_O_16_	1.658	Isomaltopaeoniflorin or isomer	–	+	–	–
P6	5.72	319.11719 [M+H]^+^	C_17_H_18_O_6_	−3.050	Paeoniflorigenone	–	+	–	+
P7	5.74	525.16095 [M+HCOO]^−^	C_23_H_28_O_11_	1.300	Albiflorin	–	+	+	+
P8	5.90	525.16089 [M+HCOO]^−^	C_23_H_28_O_11_	1.186	Isopaeoniflorin	–	+	+	–
P9	6.00	525.16095 [M+HCOO]^−^	C_23_H_28_O_11_	1.300	Paeoniflorin	–	+	+	+
P10	6.85	631.16675 [M–H]^−^	C_30_H_32_O_15_	1.590	4–O–galloylalbiflorin	–	+	-	–
P11	7.06	121.02834 [M-H]^−^	C_7_H_6_O_2_	−0.545	Benzoic acid	–	+	+	+
P12	7.30	631.16675 [M–H]^−^	C_30_H_32_O_15_	1.590	6'-O-galloylalbiflorin	–	+	–	–
P13	7.61	525.16101 [M-H]^−^	C_24_H_30_O_13_	1.414	Mudanpioside E	–	+	+	+
P14	7.64	509.16611 [M-H]^−^	C_24_H_30_O_12_	1.487	Mudanpioside D	–	+	+	+
P15	7.68	509.16617 [M+HCOO]^−^	C_23_H_28_O_10_	1.605	1-O-β-D-glucopyranosyl-8-O-benzoylpaeonisuffrone	–	+	–	–
P16	9.47	629.18790 [M+HCOO]^−^	C_30_H_32_O_12_	1.523	Benzoylpaeoniflorin	–	+	+	+
P17	9.64	629.18750 [M+HCOO]^−^	C_30_H_32_O_12_	1.618	Benzoylalbiflorin	–	+	+	–
M1	2.35	359.06229 [M-H]^−^	C_14_H_16_O_11_	3.906	Gallic acid	+Methylation +Glucuronidation	–	–	+
M2	2.49	345.0463 [M-H]^−^	C_13_H_14_O_11_	3.079	Gallic acid	+Glucuronidation	–	–	+
M3	3.68	389.14557 [M-H]^−^	C_17_H_26_O_10_	3.461	Albiflorin	–C_7_H_4_O +Methylation	–	–	+
M4	3.71	389.14554 [M-H]^−^	C_17_H_26_O_10_	3.383	Paeoniflorin	–C_7_H_4_O +Methylation	–	–	+
M5	3.84	701.18939 [M-H]^−^	C_30_H_38_O_19_	−4.229	Mudanpioside E	+Glucuronidation	+	–	–
M6	4.06	215.09155 [M+H]^+^	C_10_H_16_O_5_	0.697	Albiflorin	Deacylate albiflorin aglycone	+	–	–
M7	4.59	671.18311 [M-H]^−^	C_29_H_36_O_18_	1.966	Paeoniflorin	+Oxidation +Glucuronidation	–	–	+
M8	4.60	671.18311 [M-H]^−^	C_29_H_36_O_18_	1.966	Isopaeoniflorin	+Oxidation +Glucuronidation	–	–	+
M9	4.69	233.01230 [M-H]^−^	C_8_H_9_O_6_S	3.712	Gallic acid	+2CH_2_(Methylation) −CO_2_ +Sulfation	+	–	–
M10	4.73	373.077700 [M–H]^−^	C_15_H_18_O_11_	3.115	Gallic acid	+2CH_2_(Methylation) +Glucuronidation	+	+	+
M11	4.93	357.11923 [M–H]^−^	C_16_H_22_O_9_	3.420	Albiflorin	–C_7_H_4_O +Dehydroxylation +Oxidation	+	+	+
M12	4.96	373.07782 [M–H]^−^	C_15_H_18_O_11_	3.437	Gallic acid	+2CH_2_(Methylation) +Glucuronidation	+	+	+
M13	4.97	357.11948 [M–H]^−^	C_16_H_22_O_9_	4.120	Paeoniflorin	Pinane glucuronide	+	+	+
M14	5.00	373.07782 [M–H]^−^	C_15_H_18_O_11_	3.196	Gallic acid	+2CH_2_(Methylation) +Glucuronidation	+	+	+
M15	5.10	357.1192 [M–H]^−^	C_16_H_22_O_9_	3.336	Paeoniflorin	–C_7_H_4_O +Dehydroxylation +Oxidation	–	+	+
M16	5.24	479.11978 [M-H]^−^	C_22_H_24_O_12_	2.875	Catechin	+Methylation +Glucuronidation	+	+	+
M17	5.27	479.11966 [M-H]^−^	C_22_H_24_O_12_	2.625	Catechin	+Methylation +Glucuronidation	+	+	+
M18	5.38	373.15060 [M-H]^−^	C_17_H_26_O_9_	3.460	Albiflorin	–C_7_H_4_O _2_ + Methylation	+	+	+
M19	5.57	479.11960 [M-H]^−^	C_22_H_24_O_12_	2.499	Catechin	+Methylation	+	–	+
M20	5.91	577.12225 [M-H]^−^	C_23_H_30_O_15_S	0.144	Oxypaeoniflorin	+H_2_+Oxidation +Sulfation	–	+	–
M21	5.98	449.14517 [M-H]^−^	C_22_H_26_O_10_	2.108	Isopaeoniflorin	+Demethylation +Deoxidation	+	–	+
M22	6.01	577.12201 [M-H]^−^	C_23_H_30_O_15_S	−0.272	Oxypaeoniflorin	+H_2_+Oxidation +Sulfation	–	+	–
M23	6.56	559.11389 [M-H]^−^	C_23_H_27_O_14_S	4.091	Oxypaeoniflorin	+Sulfation	–	–	+
M24	6.80	211.09695 [M-H]^−^	C_11_H_16_O_4_	2.201	Albiflorin	–C_6_H_10_O_6_-C_7_H_5_O+ Methylation	–	–	+
M25	7.11	211.09732 [M-H]^−^	C_11_H_16_O_4_	3.953	Albiflorin	-C_6_H_10_O_6_- C_7_H_5_O+ Methylation	–	–	+
M26	7.48	199.09688 [M-H]^−^	C_10_H_16_O_4_	1.982	Paeoniflorin/albiflorin	Paeonimetabolin II	+	–	+
M27	13.49	553.18945 [M+HCOO]^−^	C_25_H_32_O_11_	−3.828	Paeoniflorin	4-O-Ethylpaeoniflorin	+	–	–

*+, Detected; –, Not Detected*.

### Putative Targets for PRA

All the identified prototypes and metabolites of PRA in rat blood samples were used for network pharmacology analysis. For the phase II metabolites, their corresponding prototypes or phase I metabolites were selected (52). The chemical structures of 31 components used for target screening were summarized in Table 3. By use of MedChem Studio, 377 putative targets for PRA were obtained as listed in Supplementary Table 1.

**Table 3:** Chemical structures of components used for target screening.

**Molecule ID**	**Molecule name**	**Structure**
–	Catechin	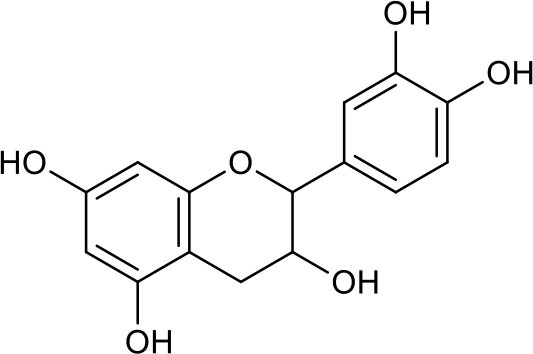
–	Gallic acid	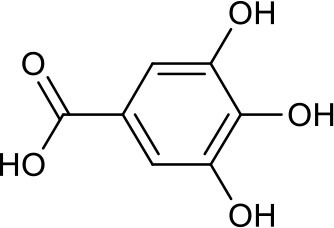
M3	–	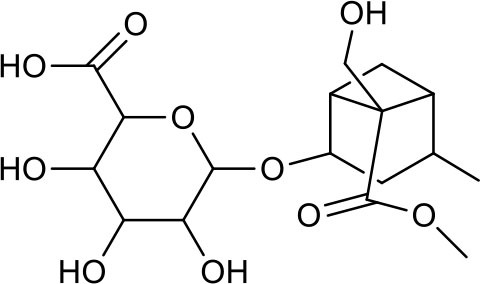
M4	–	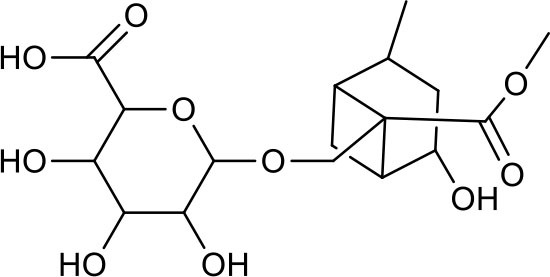
M6	-	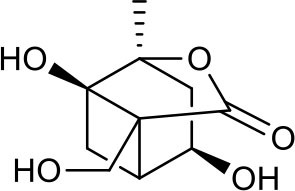
M11	–	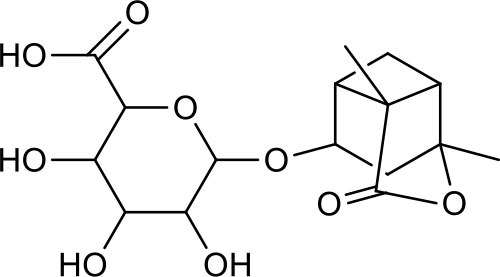
M13	–	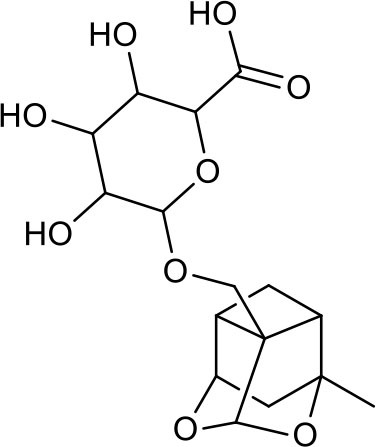
M15	–	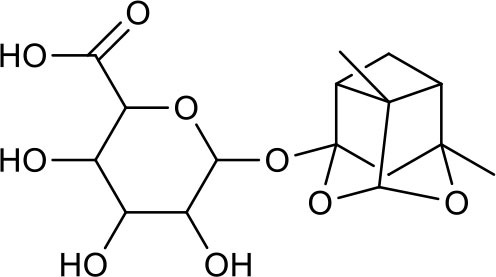
M18	–	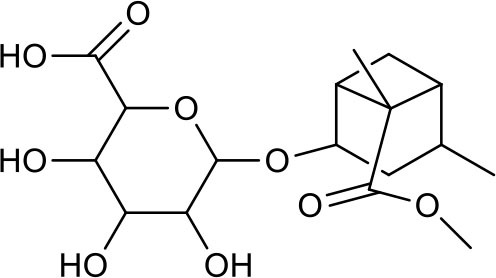
M21	–	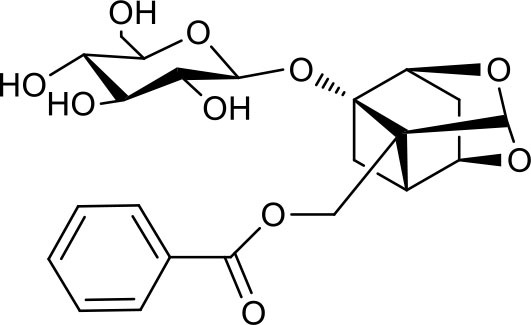
M24	–	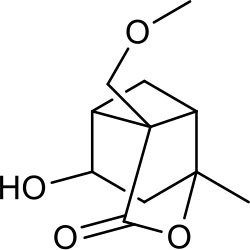
M25	–	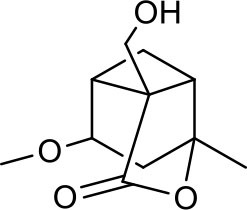
M26	–	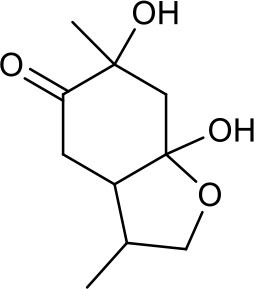
M27	–	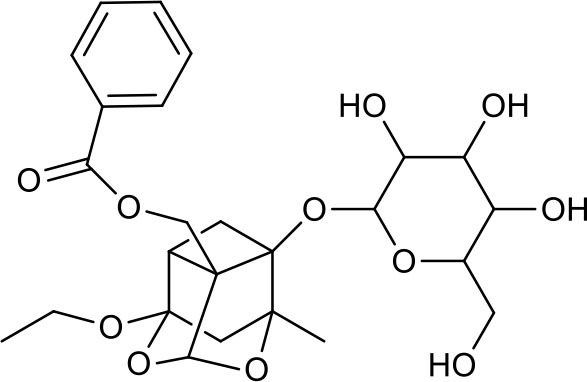
P1	3,4-Dihydroxybenzaldehyde	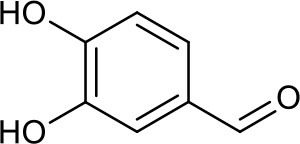
P2	ortho-Oxypaeoniflorin	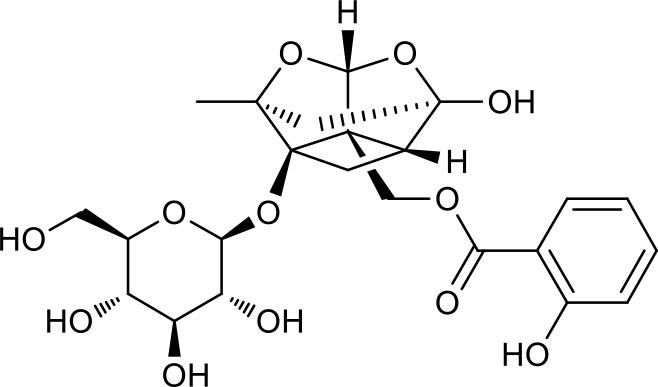
P3	Oxypaeoniflorin	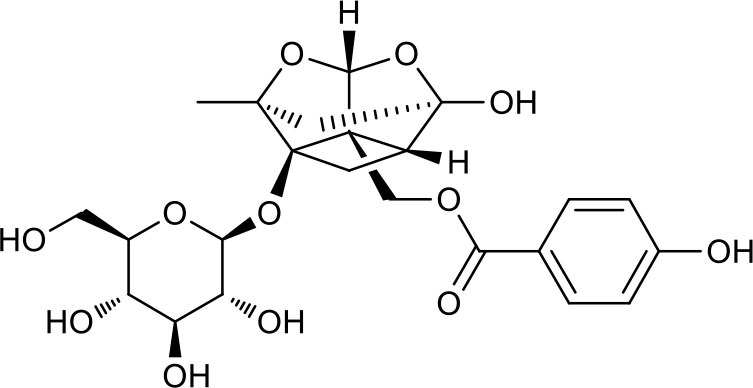
P4	Paeoniflorin sulfite	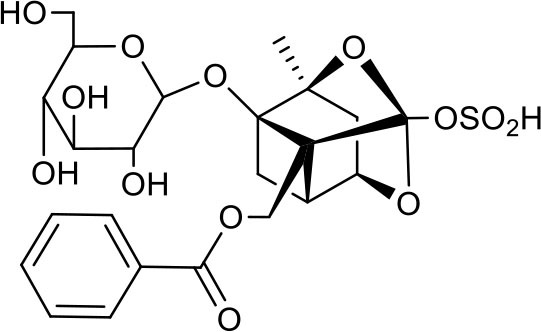
P5	Isomaltopaeoniflorin or isomer	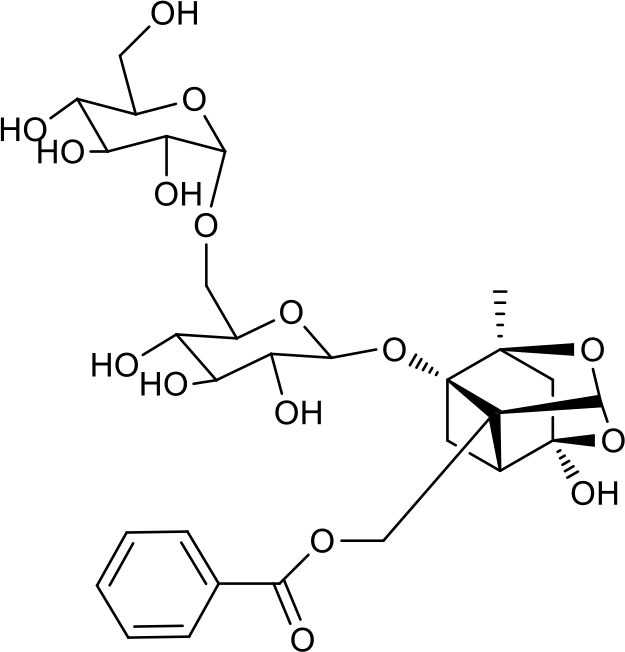
P6	Paeoniflorigenone	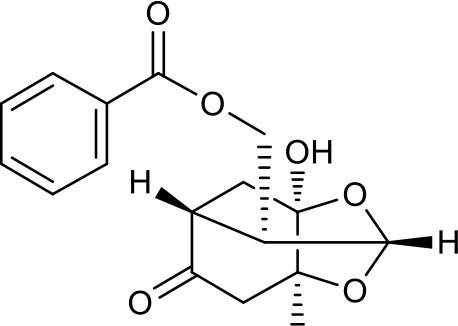
P7	Albiflorin	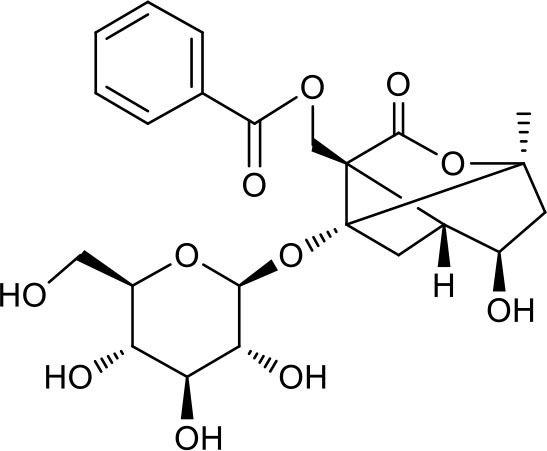
P8	Isopaeoniflorin	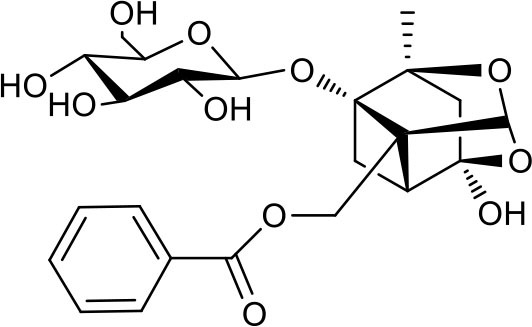
P9	Paeoniflorin	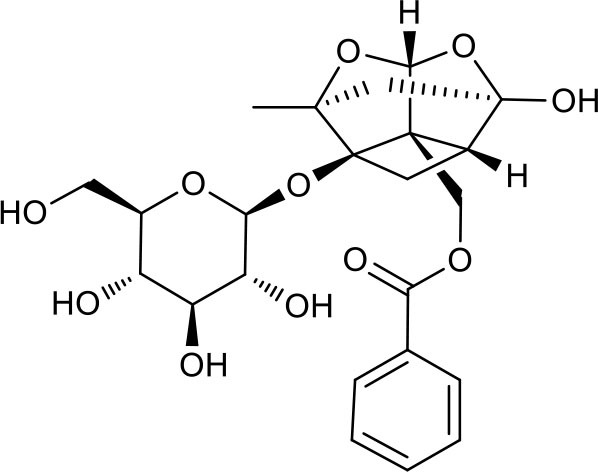
P10	4-O-galloylalbiflorin	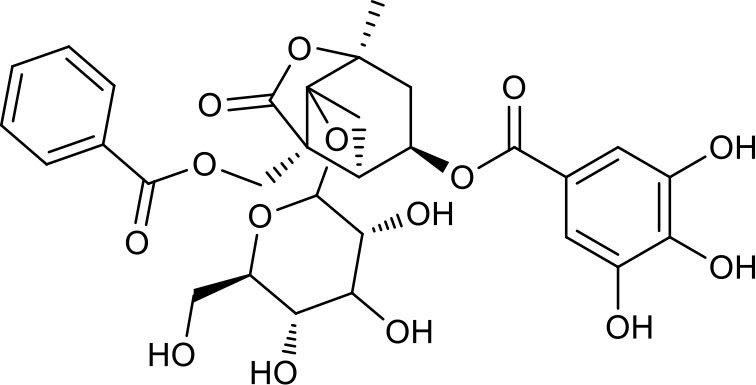
P11	Benzoic acid	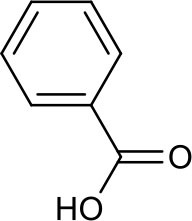
P12	6′-O-galloylalbiflorin	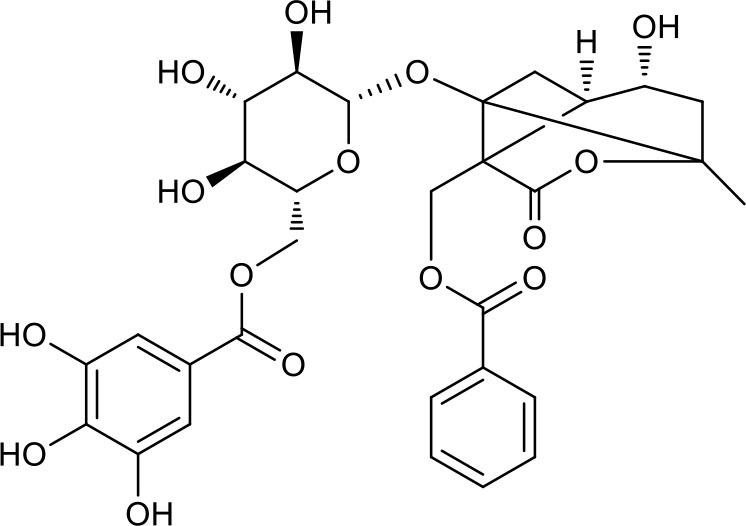
P13	Mudanpioside E	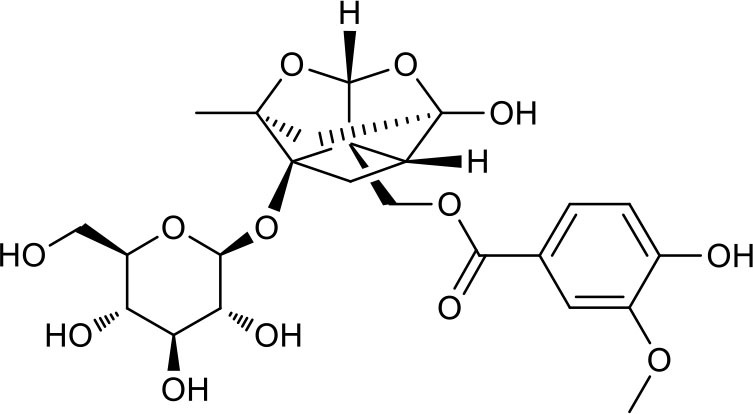
P14	Mudanpioside D	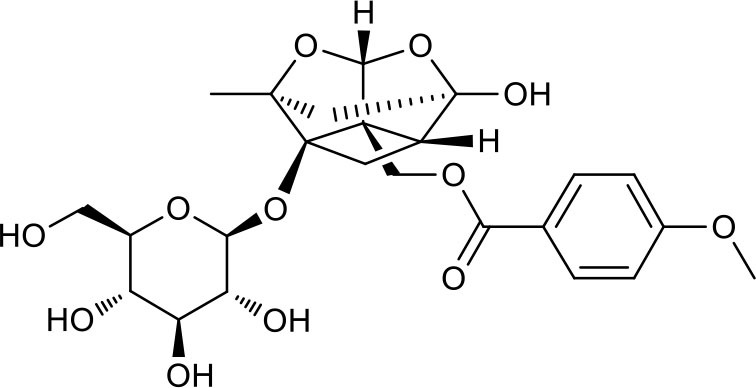
P15	1-O-β- D-glucopyranosyl -8-O-benzoylpaeonisuffrone	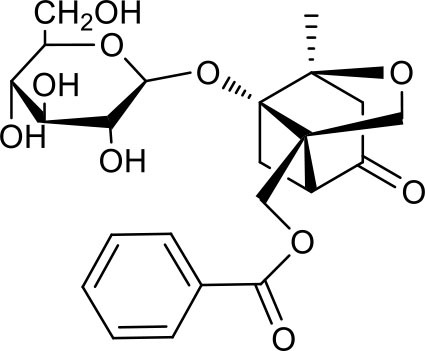
P16	Benzoylpaeoniflorin	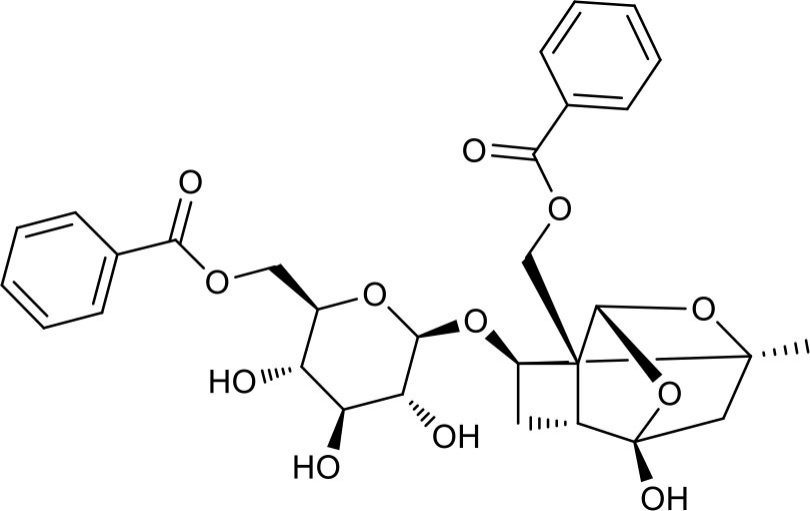
P17	Benzoylalbiflorin	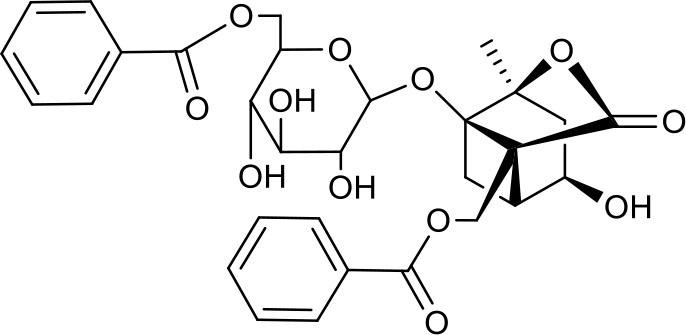

### HP-Related Targets

After eliminating redundant entries, 75 HP-related targets were acquired (Supplementary Table 2), in which 57 were from Drugbank and 18 were from OMIM.

### Network and Pathway Analysis

In order to clearly distinguish the relationship between PRA and HP, a “compound-target-disease” interaction network was constructed by connecting the major components in the blood, the PRA-associated targets as well as the HP-associated targets according to the PPI data from the STRING database. In the PPI network, the node with a higher score may be more important in the central correlation. Therefore, 408 nodes and 4,073 edges, ranked by the scores (>0.4), were included in the network (Supplementary Table 3).

To identify the nodes which were highly interconnected within the network, the DC, BC, and CC values of all nodes in the drug target-disease network were calculated. According to the screen condition, 133 hubs were considered as the critical targets accordingly (DC > 16, BC > 0.0013, and CC > 0.3882). Among them, 111 hubs were PRA-associated targets, 10 hubs were HP-associated targets, and 12 hubs were both associated with PRA and HP. The details were listed in Supplementary Table 4.

**Figure 3:**
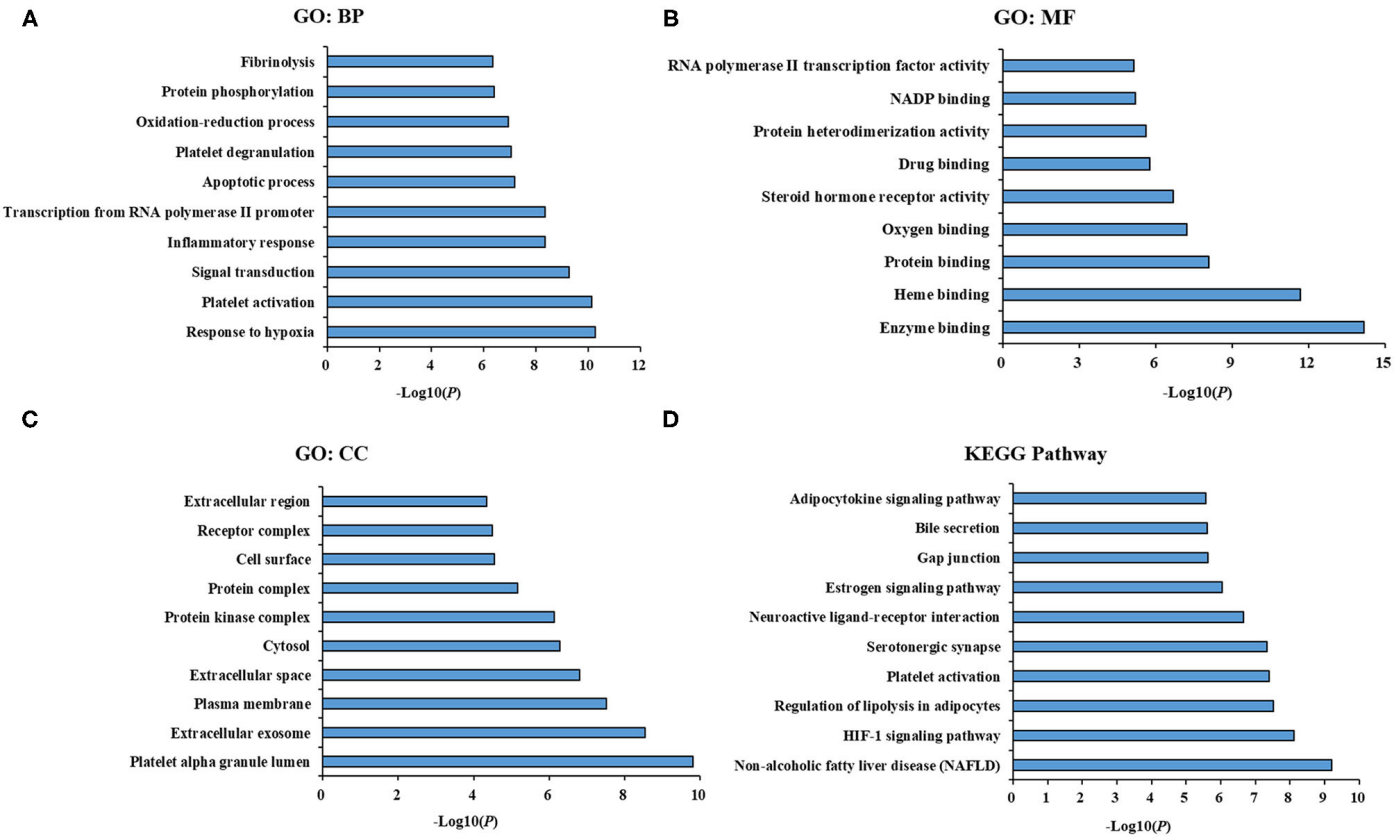
GO term performance and pathway enrichment analysis of major hubs. **(A)** GO term performance by a biological process (BP); **(B)** GO term performance by a molecular function (MF); **(C)** GO term performance by a cellular component (CC); **(D)** pathway enrichment analysis by KEGG. The ordinate stands for GO terms or main pathways, the primary abscissa stands for minus log10(*P*).

To understand the biological functions, pathways or cell localization of the major hubs, GO enrichment analysis was performed, including biological process (BP), cell component (CC), and molecular function (MF). The results indicated that hundreds of GO entries were enriched, and the top 10 significant entries in the BP, MF, and CC categories were listed in Figures 3A–C. The enriched BP ontologies were dominated by response to hypoxia, platelet activation, signal transduction, inflammatory response, transcription from RNA polymerase II promoter, apoptotic process, platelet degranulation, oxidation-reduction process, protein phosphorylation, fibrinolysis (Figure 3A). And the enriched MF ontologies were dominated by enzyme binding, heme binding, protein binding, oxygen binding, steroid hormone receptor activity, drug binding, protein heterodimerization activity, NADP binding, protein heterodimerization activity, chromatin binding (Figure 3B). Furthermore, platelet alpha granule lumen, extracellular exosome, plasma membrane, extracellular space, cytosol, protein kinase complex, protein complex, cell surface, receptor complex, and extracellular region were ranked as top 10 CC ontologies (Figure 3C).

To further excavate the significance of the major hubs, KEGG pathway enrichment analysis were conducted and resulted in 108 pathways with significant enrichment (*P* < 0.05). The top 10 pathways were listed in Figure 3D, which could be categorized into three major functional groups: metabolism (such as non-alcoholic fatty liver disease, regulation of lipolysis in adipocytes, estrogen signaling pathway, adipocytokine signaling pathway), blood circulation system (such as HIF-1 signaling pathway, platelet activation and serotonergic synapse), and signal transduction (such as neuroactive ligand-receptor interaction, gap junction and bile secretion).

**Figure 4:**
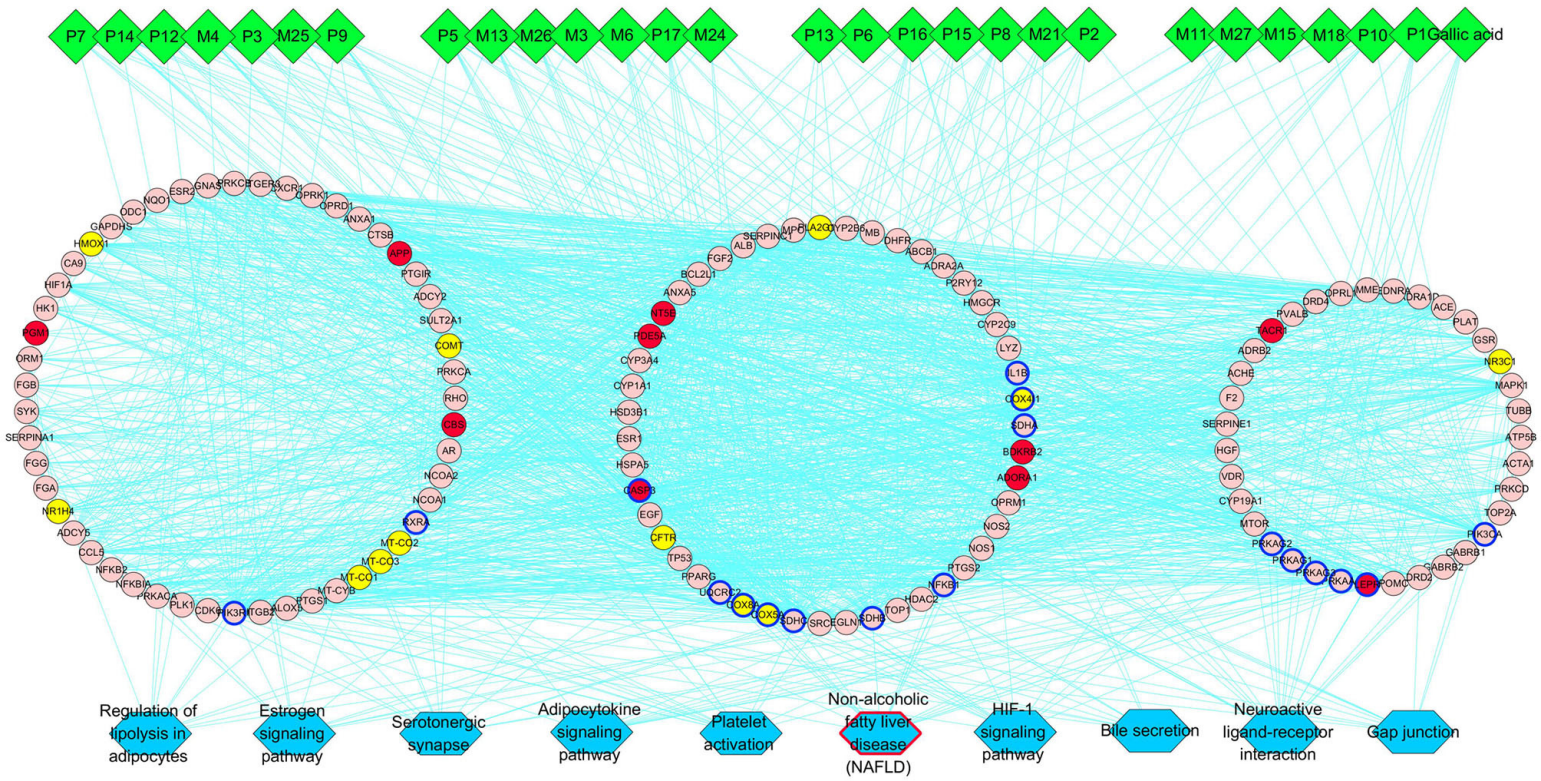
PRA key components-major hubs–main pathways network. Green diamonds represent each component (prototype/metabolite) in PRA; round pink nodes represent putative targets of PRA components; round red nodes represent known therapeutic targets for HP; round yellow nodes represent both putative targets of PRA components and known therapeutic targets for HP, and targets circled in blue represent those important hubs involved in the progression of PRA acting on HP; blue rectangles represent top 10 pathways from enrichment analysis of major targets, and blue rectangle circled in red represent the most significant pathway; edges represent interactions among PRA key components, putative targets, known therapeutic targets for the treatment of HP, and main pathways.

Then we built a network which connects the interaction among the components of PRA in the blood, main hubs, and significant pathways to achieve a comprehensive understanding of the action mechanism (Figure 4). Interestingly, the pathway of non-alcoholic fatty liver disease (NAFLD) was highly enriched in main KEGG pathways, suggesting that PRA might provide bright prospects for the prevention and treatment of NAFLD.

### Potential Mechanisms of PRA in Treating NAFLD

**Figure 5:**
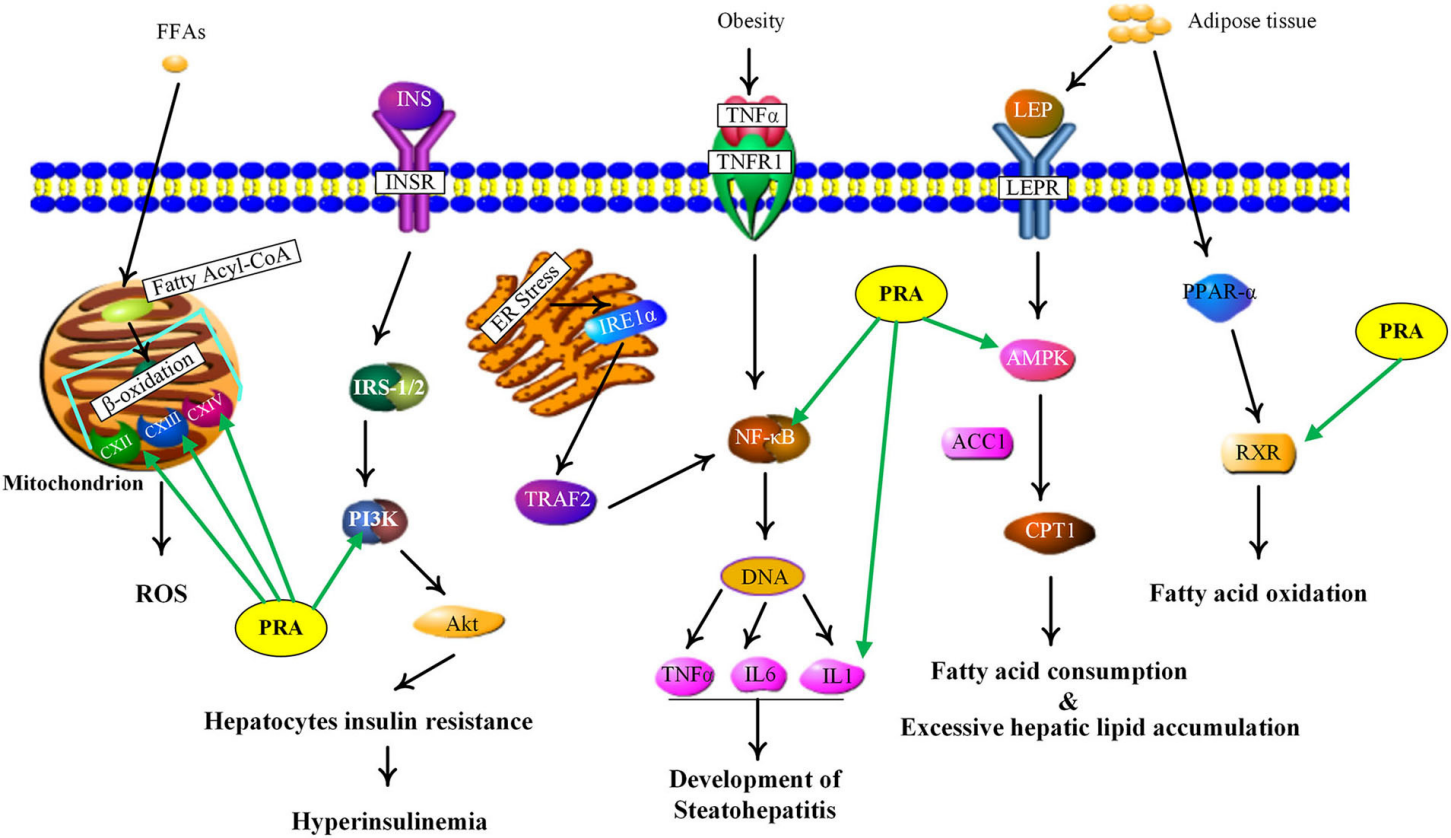
NAFLD signaling pathway influenced by major putative targets of PRA components.

NAFLD, defined as excessive hepatic fat accumulation in the absence of alcohol consumption (53), has become the leading cause of chronic liver disease worldwide (54). Pathologically, NAFLD includes several subgroups, such as simple steatosis, non-alcoholic steatohepatitis (NASH) and non-alcoholic steatofibrosis (NASF), which may progress to irreversible cirrhosis and hepatocellular carcinoma (55, 56). As shown in Supplementary Table 5, the PRA putative targets involved in NAFLD include adenosine 5'-monophosphate (AMP)-activated protein kinases (AMPKs, including PRKAG1, PRKAG2, PRKAG3, and PRKAA1), retinoid X receptor alpha (RXRA), nuclear factor kappa B subunit 1 (NFKB1), interleukin 1 beta (IL1B), mitochondrial electron transport chain complexes (UQCRC2, COX8A, COX4I1, COX5A, SDHA, SDHB, and SDHC), phosphoinositide-3-kinase (PI3K, including PIK3CA and PIK3R1). Figure 5 showed the main NAFLD pathogenesis concerning the PRA putative targets and the details were discussed below.

Current evidence indicates that adipose tissue inflammation could drive the progression of NAFLD in obesity (57). Leptin and adiponectin, two important adipokines secreted from adipose tissue, could cause the phosphorylation and activation of AMPK via binding with their corresponding receptors, called leptin receptor (LEPR) and adiponectin receptor (ADIPOR) (58). AMPK is a highly conserved master regulator of energy metabolism in the liver and its activation would lead to fatty acid oxidation, the increase of glucose uptake and the suppression of lipogenesis through multiple metabolic pathways (59). However, inactivated AMPK was observed in NAFLD, which triggers the inhibition of carnitine palmitoyl transferase 1 (CPT1) via dephosphorylating acetyl-CoA carboxylase 1 (ACC1), leading to the reduction of fatty acid consumption and excessive hepatic lipid accumulation (56). Therefore, AMPK activation could be a potential candidate for treating NAFLD. According to our predicted results, the PRA constituents targeting AMPK are two phenolics, including gallic acid and 3,4-dihydroxybenzaldehyde (P1), which are well-known natural activators of AMPK. For example, gallic acid has been reported to exert beneficial effects on body weight and glucose homeostasis via AMPK activation (60). 3,4-dihydroxybenzaldehyde has also been demonstrated to inhibit the activity of glucose-6-phosphatase through stimulating AMPK phosphorylation (61). Taken together, the anti-NAFLD function of PRA may be partially attributed to the activation of AMPKs (PRKAG1, PRKAG2, PRKAG3, and PRKAA1).

RXR, an obligate heterodimeric partner of many nuclear receptors (NR), occupies a central place in NR signaling and plays a critical role in maintaining energy homeostasis (62). In human liver, RXR could promote fatty acid oxidation by forming a heterodimer with peroxisome proliferator-activated receptor alpha (PPARα) which could be activated by adiponectin to magnify this signaling (63). According to our predicted results, nine compounds (P2, P5, P8, P9, P12, P13, P16, P17, and M27) were considered as RXR agonists and they may be partially responsible for the anti-NAFLD effect of PRA.

NF-κB, an integrator of inflammatory pathway networks, is also essential in the occurrence and development of NAFLD (64). In response to endoplasmic reticulum (ER) stress, serine/threonine-protein kinase/endoribonuclease (IRE1) is activated, which sequentially triggers the activation of tumor necrosis factor (TNF) receptor-associated factor 2 (TRAF2) and NF-κB, leading to the expression and release of inflammatory cytokines (such as IL-1, IL-6, and TNF-α) via binding to DNA. Additionally, in response to obesity, transduction of TNF-α signal by TNF receptor superfamily member 1A (TNFR1) also involves activation of NF-κB (65, 66). Hence, aberrant activation of NF-κB is one of the major risk factors in the pathological process of steatohepatitis. It has been evident that P1 and gallic acid could alleviate inflammation through the down-regulation of NF-κB (67, 68). Therefore, NF-κB can be speculated as the potential therapeutic target of PRA in NAFLD.

Mitochondria, whose most important function is to generate adenosine triphosphate (ATP), plays a fundamental role in lipid metabolism and oxidative stress (69). Upon entering the mitochondria, free fatty acids (FFAs) are converted into fatty acyl-CoA and then undergo the process of β-oxidation, which is catalyzed by a series of mitochondrial enzymes and generates acetyl-coA, NADH (nicotinamide adenine dinucleotide) and FADH2 (flavine adenine dinucleotide, reduced) (70). NADH and FADH2 could transfer their electrons to the electron transport chain (ETC) to induce an oxidation-reduction reaction at each step and ATP will be produced by oxidative phosphorylation, a process which needs the action of five ETC complexes (71, 72). However, impaired electron transport within the ETC induces electron leakage from the ETC complex, thus resulting in reactive oxygen species (ROS) production. The increased oxidative stress would induce the release of inflammatory cytokines and affect the activity of major enzymes associated with lipid metabolism, which may contribute to the pathological process of NAFLD (73). Many components have been shown to play antioxidative roles by reducing ROS levels, such as paeoniflorin (74) and albiflorin (75). Therefore, PRA may modify the NAFLD status by decreasing ROS formation and ameliorating mitochondrial dysfunction.

PI3K, a member of lipid kinases family, is associated with an extraordinarily diverse group of cellular functions (76). In human liver, transduction of insulin signal by insulin receptor (InsR) involves activation of insulin receptor substrate-1/2 (IRS-1/2), which in turn activates PI3K, leading to the activation of protein kinase B (Akt). Finally, the activated Akt mediates the diverse pharmacological functions (like glucose uptake and lipid metabolism) of insulin via transmitting the biological signal to the downstream targets (77, 78). An increasing body of evidence suggests that the deregulation of PI3K/Akt signaling pathway in hepatocytes may lead to insulin resistance and further induce NAFLD development (77). The two abundant components in PRA, albiflorin, and paeoniflorin, have been reported to exert beneficial effects on NAFLD and obesity via regulating PI3K/Akt pathway (79, 80). Therefore, the restoration of PI3K activity may be involved in the anti-NAFLD function of PRA.

Taken together, PRA might exert curative effects on NAFLD through acting on multiple targets involved in multiple signaling pathways. The results also pointed out the huge potential for the clinical use of MEP with heptoprotective activity for preventing and treating NAFLD.

### Molecular Docking

Molecular docking studies were used to verify the interactions between the PRA components and the targets related to NAFLD (PRKAG3, UQCRC2, RXRA, PRKAG1, COX8A, PRKAG2, COX4I1, NFKB1, COX5A, SDHA, SDHB, SDHC, IL1B, PIK3CA, PRKAA1, and PIK3R1). The results indicated that the PRA components had been docked successfully with PRKAG1, PRKAA1, NFKB1, PIK3CA, PIK3R1, SDHC, and COX4I1, as listed in Supplementary Table 6. Therefore, PRA may treat NAFLD mainly through these seven targets.

### SPR Assays for Affinity

**Figure 6:**
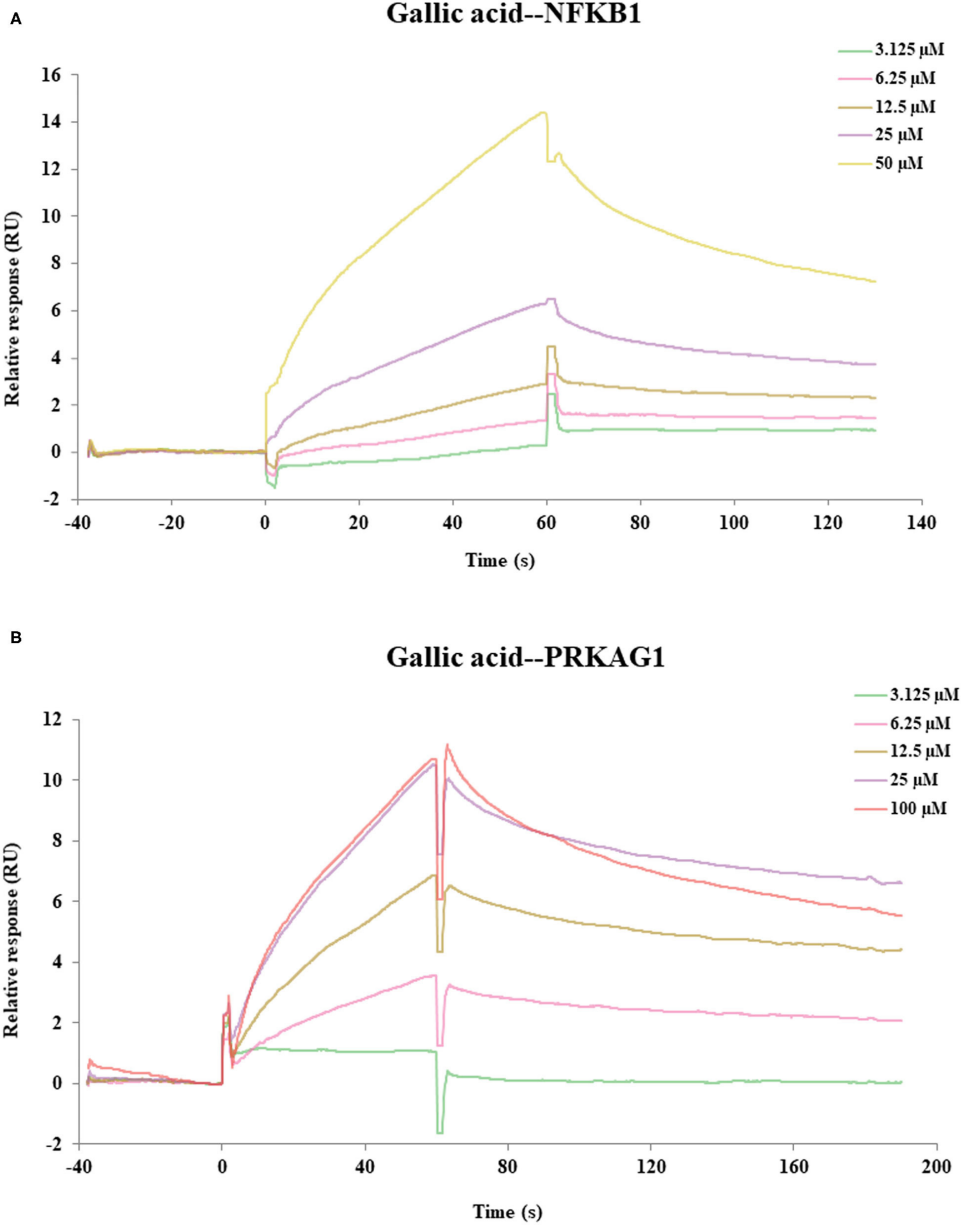
The SPR assay of the interaction of gallic acid with NFKB1, gallic acid with PRKAG1. **(A)** The SPR curves of gallic acid binding to NFKB1. **(B)** The SPR curves of gallic acid binding to PRKAG1.

SPR assay was used to verify the direct binding activities of the target proteins and their corresponding compounds according to the results of molecular docking, including gallic acid-PRKAG1, 3,4-Dihydroxybenzaldehyde-PRKAG1, gallic acid-NFKB1, 3,4-Dihydroxybenzaldehyde-NFKB1. As depicted in Figure 6, we found two compound–target pairs that exhibited relatively better affinities, including gallic acid-NFKB1 (K_D_ = 28.6 μM) and gallic acid-PRKAG1 (K_D_ = 14 μM). Interestingly, gallic acid had relatively slow association and dissociation rates at NFKB1 and PRKAG1 (81), suggesting that gallic acid may be a promising ligand for *in vivo* targeting NFKB1 and PRKAG1. The above results demonstrated the validity of network pharmacology and molecular docking approaches, and the in-depth biological functions of these ligand–target pairs were worth exploring.

## Conclusions

Compared with conventional metabolite identification method for partial metabolism in the whole metabolic route, we developed a sequential metabolites identification approach by integrating IPVS and LC/MS to characterize the dynamic biotransformation process of MEP. Using this strategy, the metabolic profile of PRA was described rapidly and comprehensively, including 17 prototypes and 27 metabolites, which were bio-transformed via oxidation, methylation, sulfation, and glucuronidation. Next, the network and pathway analysis were performed based on the identified metabolites and the results indicated that the pathway of non-alcoholic fatty liver disease (NAFLD) was highly enriched and 16 key targets were found, suggesting that PRA may have beneficial effects in the prevention and treatment of NAFLD. Moreover, the molecular docking and SPR experiments showed that several constituents exhibited good affinity to specific targets (gallic acid-NFKB1, K_D_ = 28.6 μM; gallic acid-PRKAG1, K_D_ = 14 μM). Collectively, this work provides a systems perspective to study the chemical and functional basis of PRA for preventing and treating liver disease, which demonstrated that the proposed strategy may be a powerful tool in screening potential active compounds and their corresponding targets from other MEPs.

## Data Availability Statement

The original contributions presented in the study are included in the article/Supplementary Material, further inquiries can be directed to the corresponding author/s.

## Ethics Statement

The animal study was reviewed and approved by the Animal Ethnic Committee of Beijing University of Chinese Medicine.

## Author Contributions

YL, GY, ZL, and YS conceived and designed the experiments. ZL, XH, and WY performed the experiments. GW, JW, XJ, MS, and XL contributed to data analysis and manuscript preparation. ZL and GY wrote the paper. All authors have read and approved the final manuscript.

## Funding

This study was supported by the National Key Research and Development Program of China (No. 2019YFC1710105), the National Traditional Chinese Medicine Standardization Project (ZYBZH-Y-HUB-20), and National Natural Science Foundation of China (No. 81973295).

## Conflict of Interest

GW was employed by company Zhongcai Health (Beijing) Biological Technology Development Co., Ltd. The remaining authors declare that the research was conducted in the absence of any commercial or financial relationships that could be construed as a potential conflict of interest.

## Publisher's Note

All claims expressed in this article are solely those of the authors and do not necessarily represent those of their affiliated organizations, or those of the publisher, the editors and the reviewers. Any product that may be evaluated in this article, or claim that may be made by its manufacturer, is not guaranteed or endorsed by the publisher.
