# Supplementary material for: Mechanism of Paeoniae Radix Alba in the Treatment of Non-alcoholic Fatty Liver Disease Based on Sequential Metabolites Identification Approach, Network Pharmacology, and Binding Affinity Measurement
Source: Front Nutr. 2021 Sep 16;8:677659. doi: 10.3389/fnut.2021.677659 (PMC8481579; doi:10.3389/fnut.2021.677659)
Supplement: Supplementary file 7 [file Data_Sheet_1.PDF]

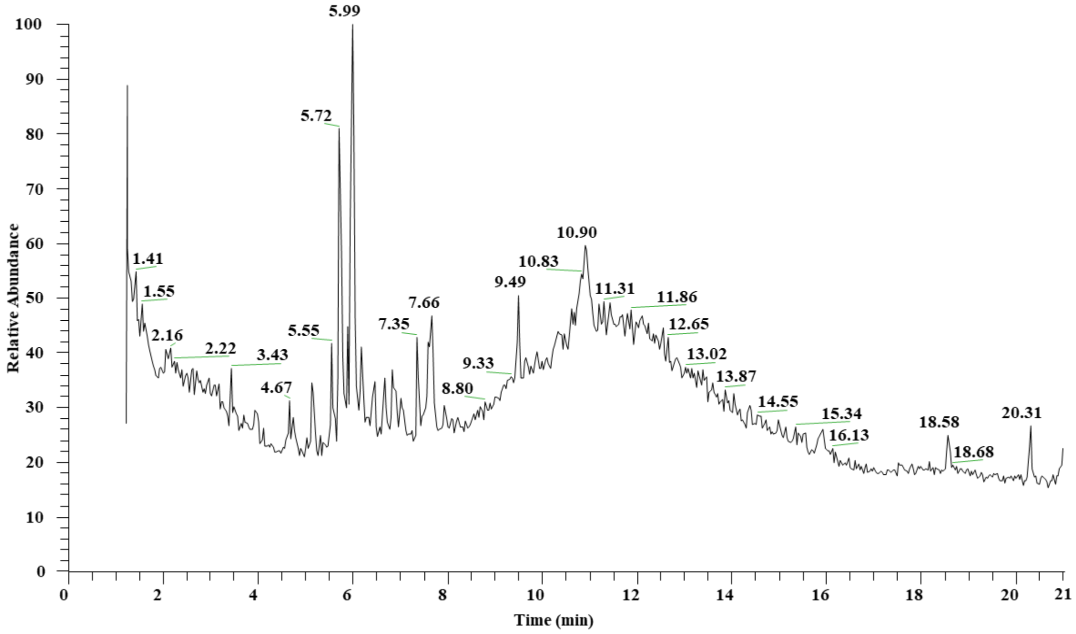

Fig. S1. Total ion chromatogram of the water extract of PRA in positive ion mode

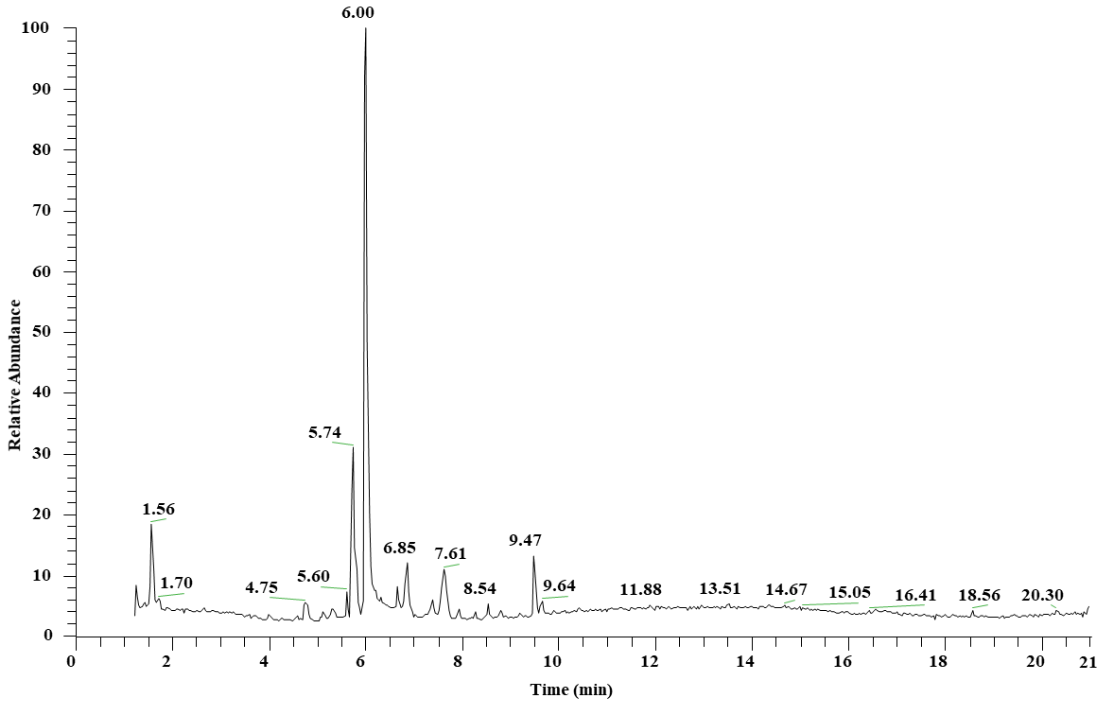

Fig. S2. Total ion chromatogram of the water extract of PRA in negative ion mode

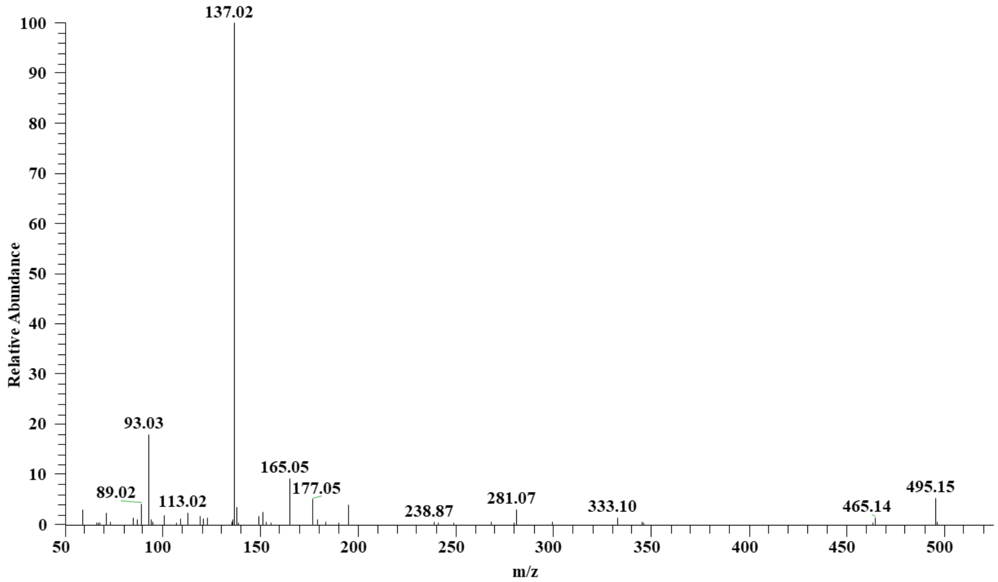

(A)

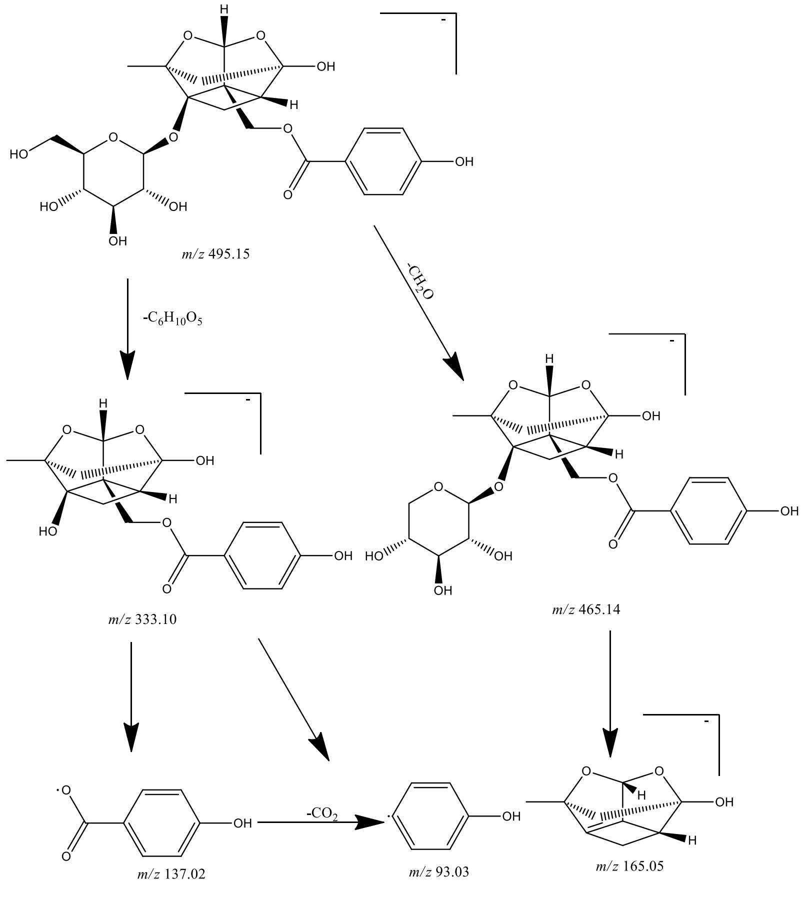

(B)

Fig. S3. (A) Mass spectra and (B) proposed fragmentation pathways of oxypaeoniflorin

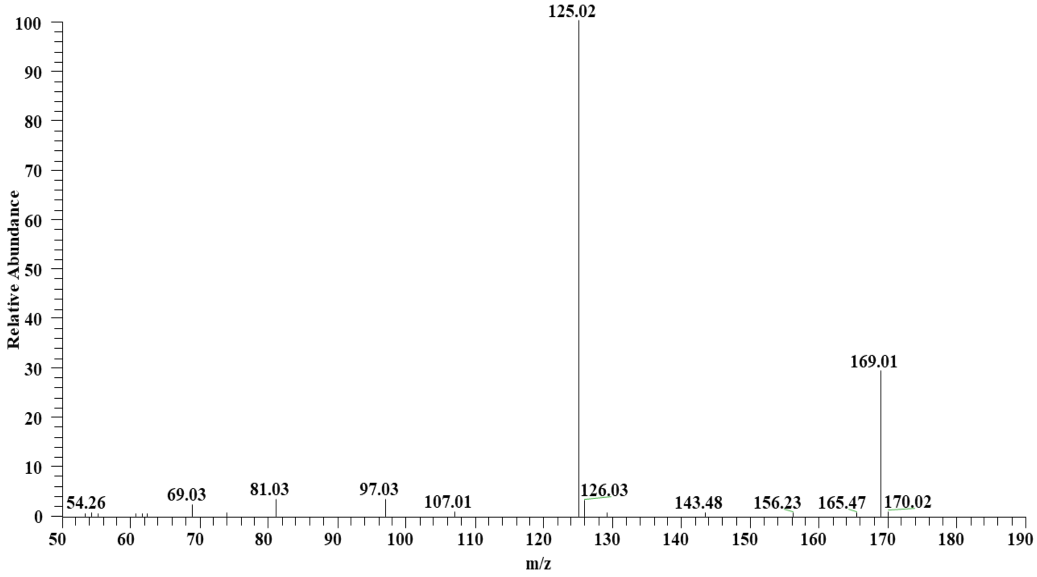

(A)

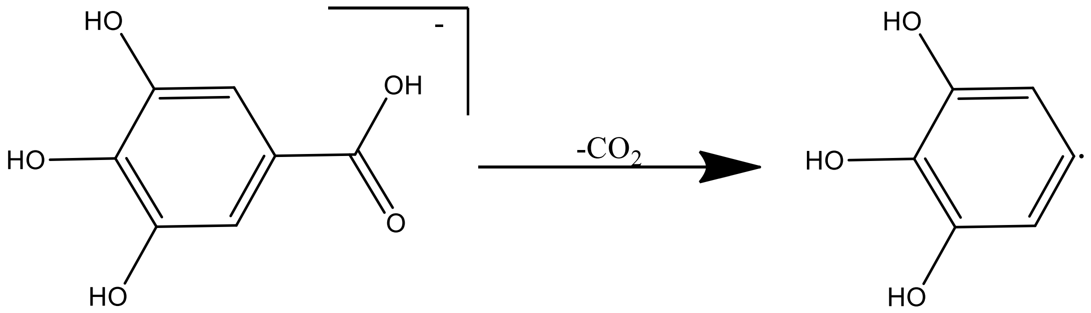

(B)

Fig. S4. (A) Mass spectra and (B) proposed fragmentation pathways of gallic acid

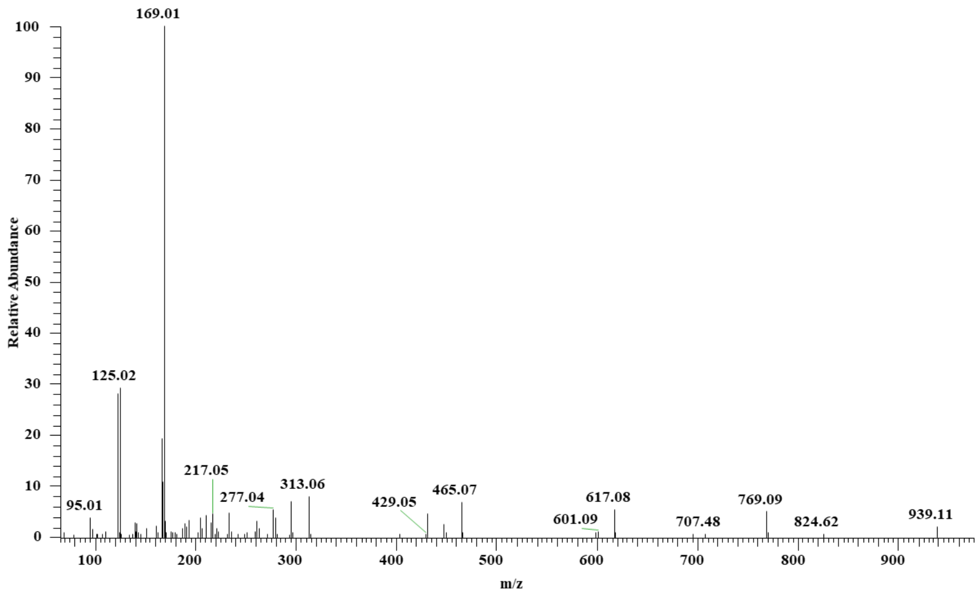

(A)

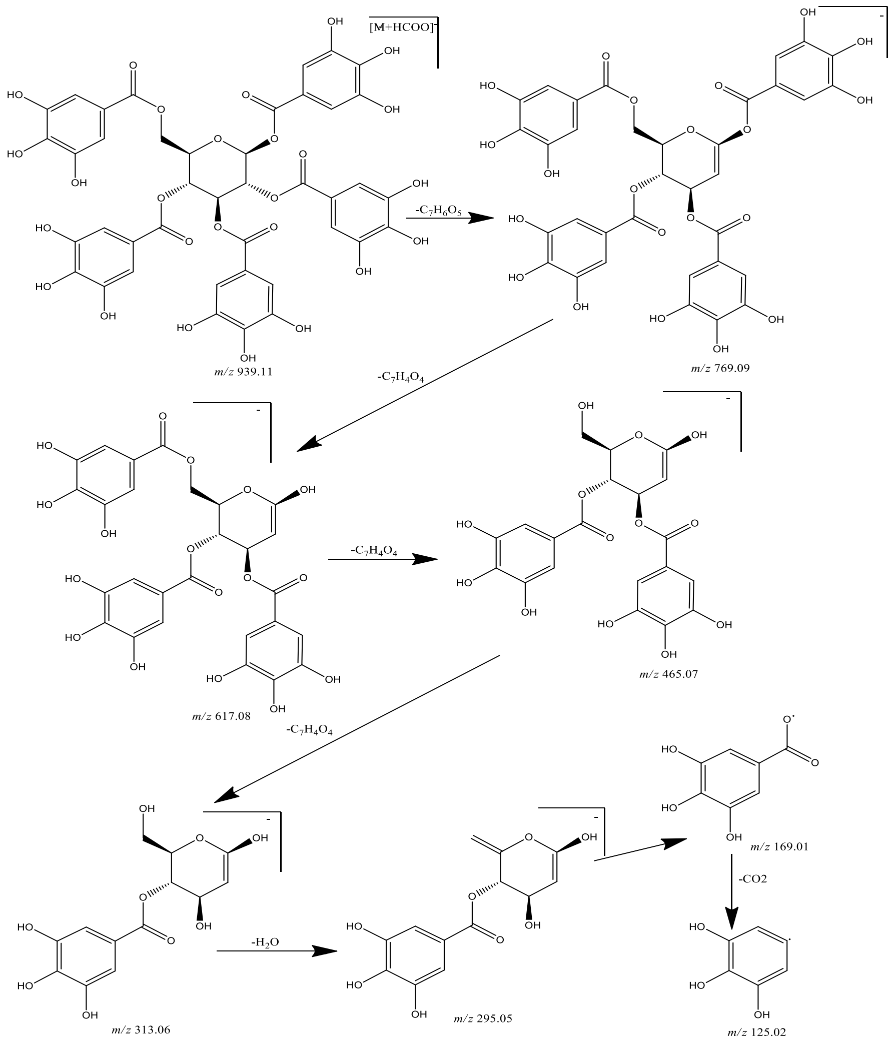

(B)

Fig. S5. (A) Mass spectra and (B) proposed fragmentation pathways 1,2,3,4,6-penta-O-galloyl-β-D-glucose

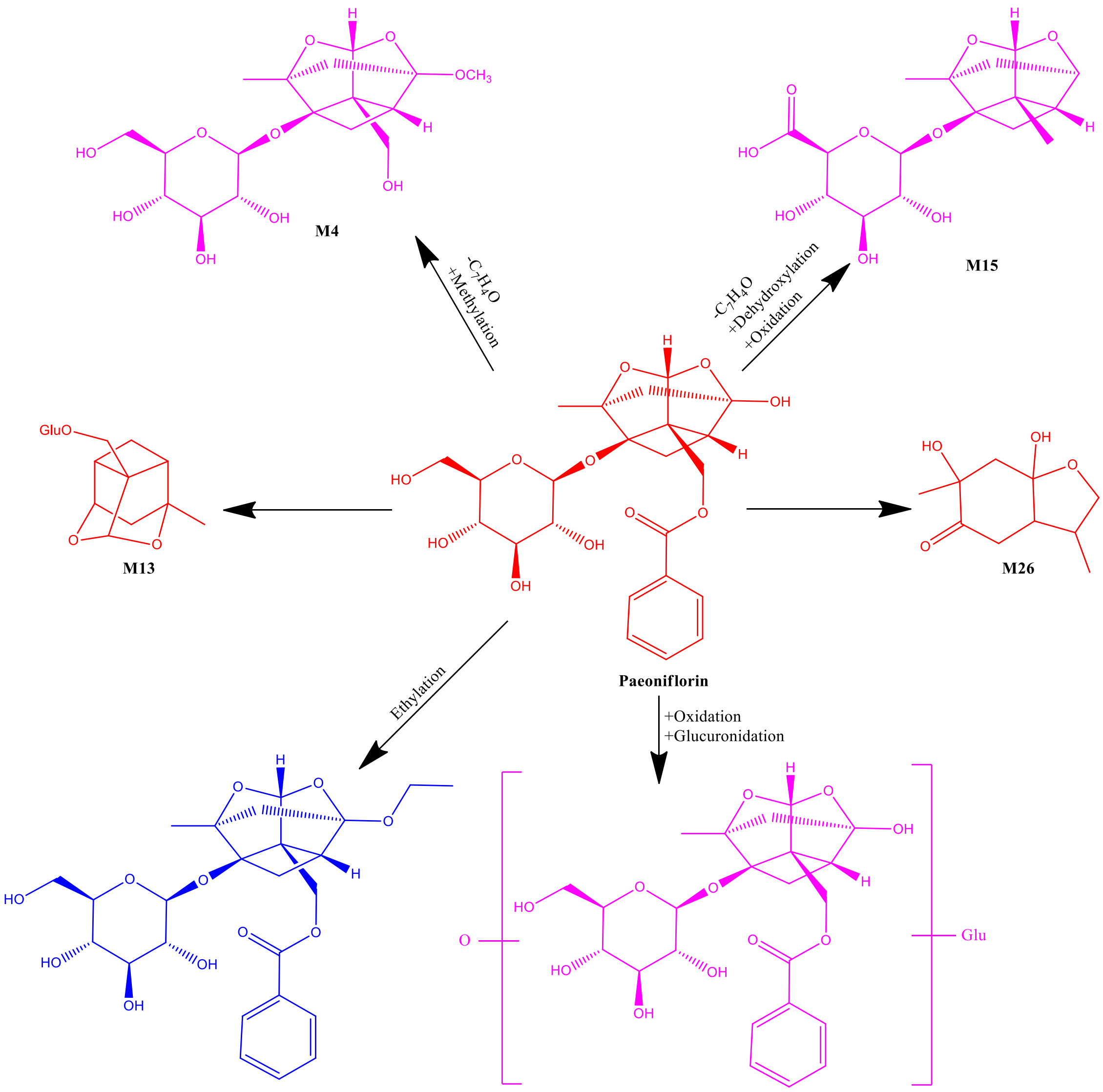

Fig. S6. The proposed metabolic pathways of paeoniflorin

The compounds could only be detected in mesenteric blood were colored in blue; the compounds could only be detected in systemic blood stream were colored in pink; the compounds could both be detected in mesenteric blood and systemic blood stream were colored in red.

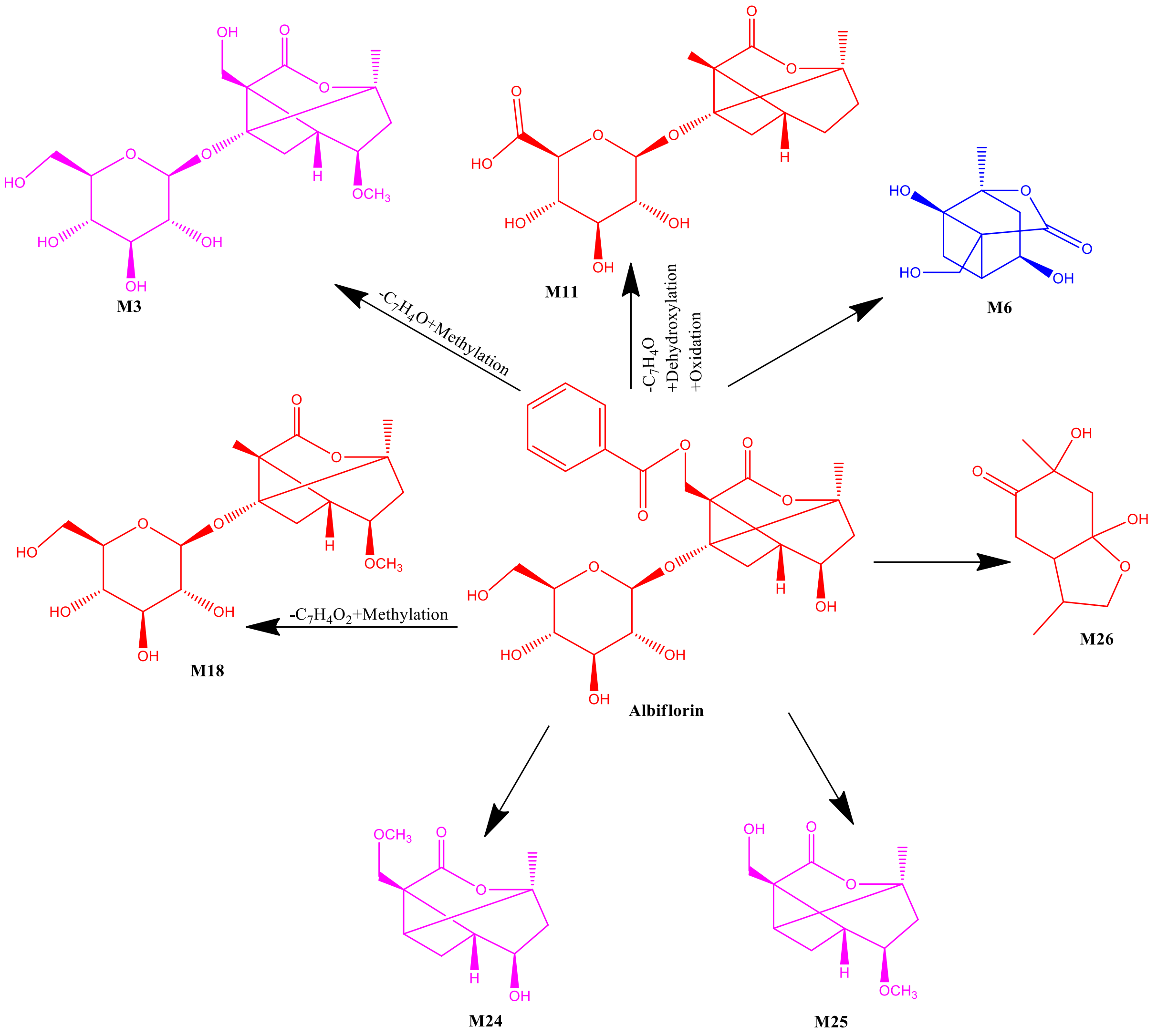

Fig. S7. The proposed metabolic pathways of albiflorin

The compounds could only be detected in mesenteric blood were colored in blue; the compounds could only be detected in systemic blood stream were colored in pink; the compounds could both be detected in mesenteric blood and systemic blood stream were colored in red.

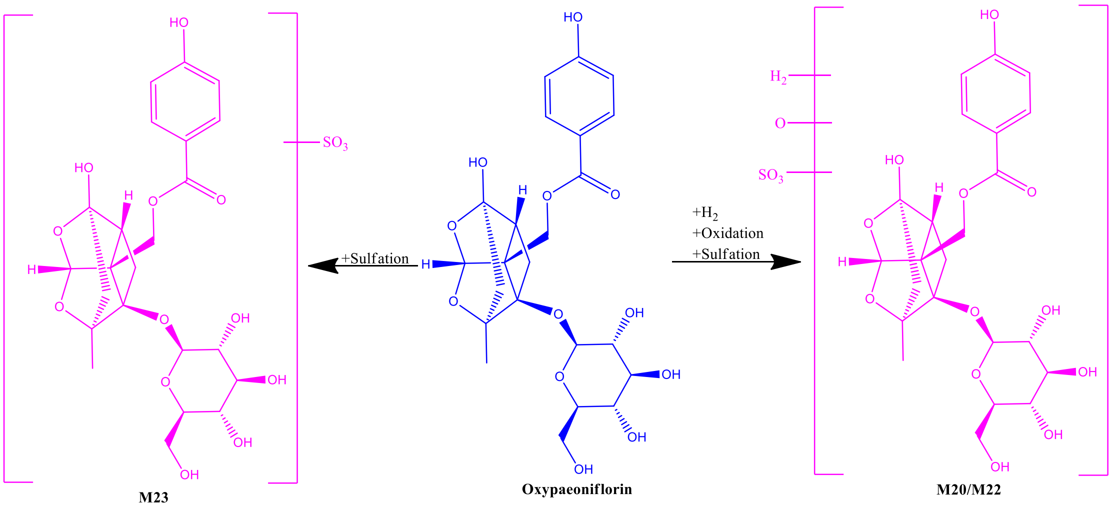

Fig. S8. The proposed metabolic pathways of oxypaeoniflorin

The compounds could only be detected in mesenteric blood were colored in blue; the compounds could only be detected in systemic blood stream were colored in pink.

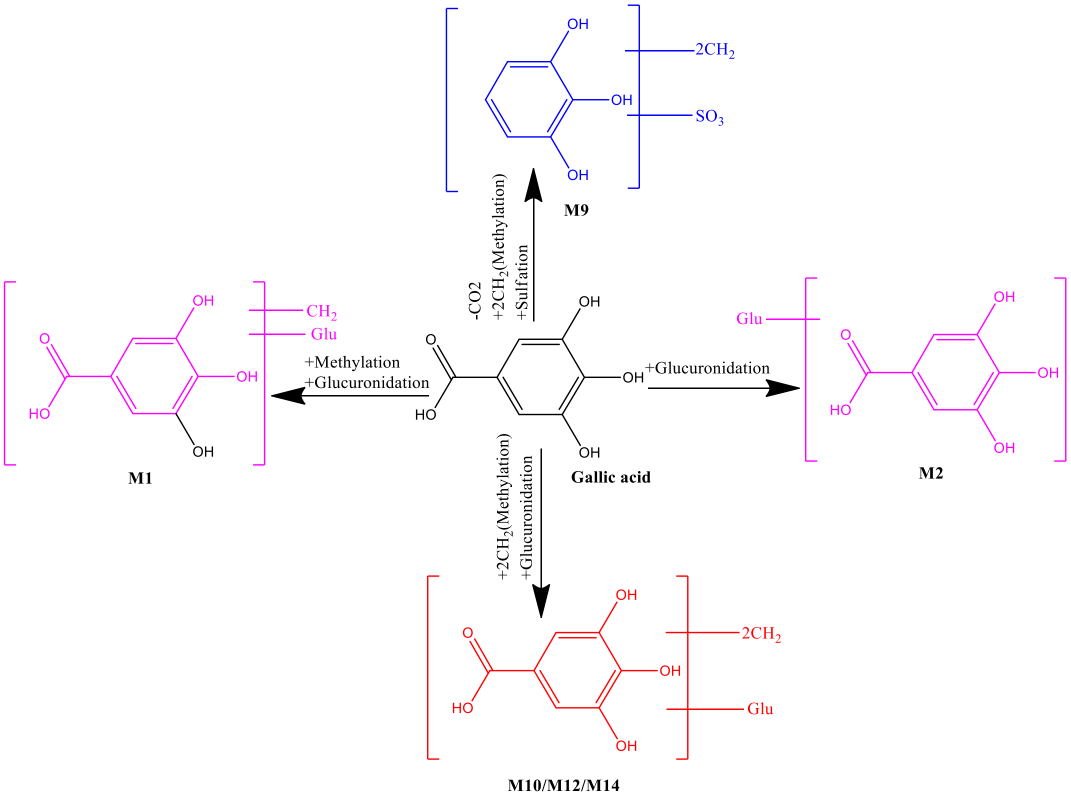

Fig. S9. The proposed metabolic pathways of gallic acid

The compounds could only be detected in mesenteric blood were colored in blue; the compounds could only be detected in systemic blood stream were colored in pink; the compounds could both be detected in mesenteric blood and systemic blood stream were colored in red.

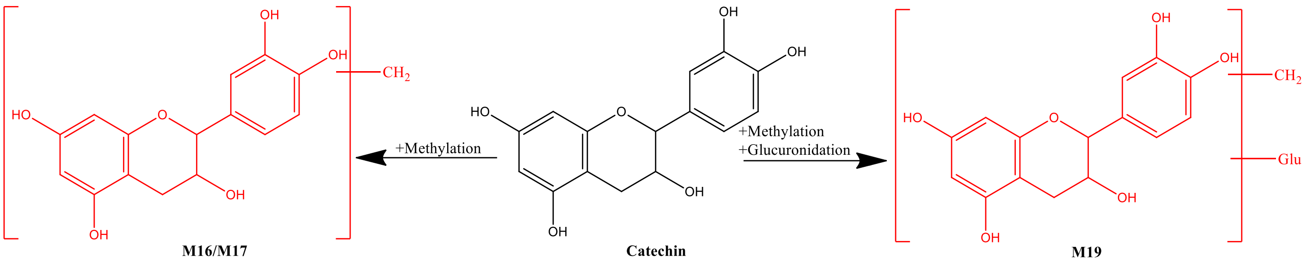

Fig. S10. The proposed metabolic pathways of catechin

The compounds could not be detected in any blood samples were colored in black; the compounds could both be detected in mesenteric blood and systemic blood stream were colored in red.

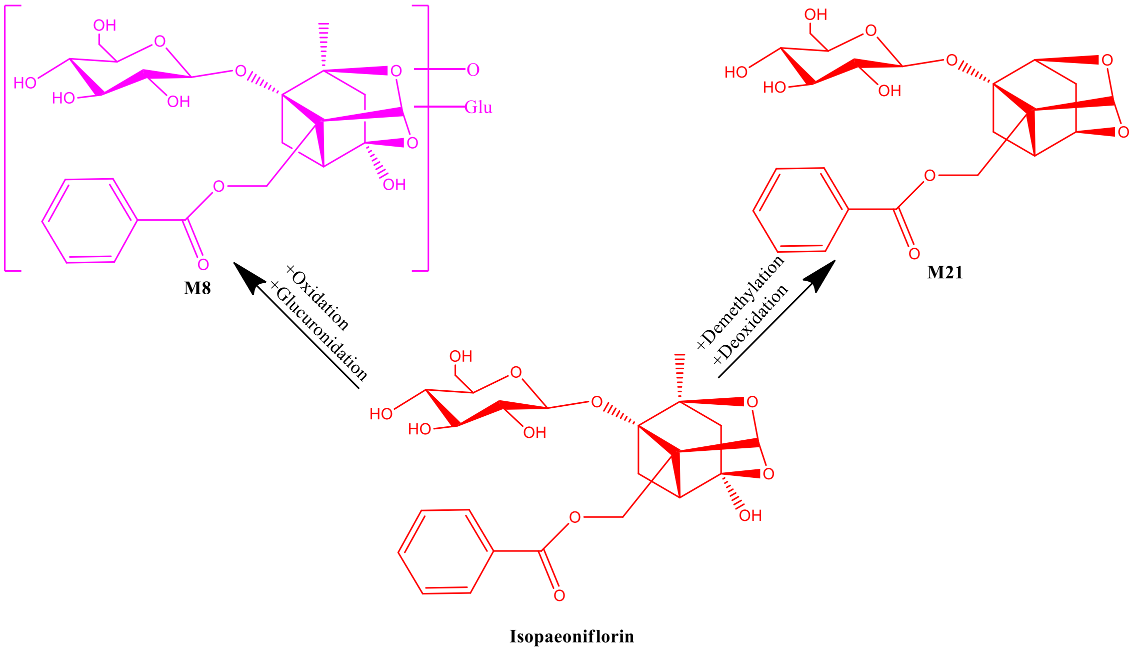

Fig. S11. The proposed metabolic pathways of isopaeoniflorin

The compounds could only be detected in systemic blood stream were colored in pink; the compounds could both be detected in mesenteric blood and systemic blood stream were colored in red.

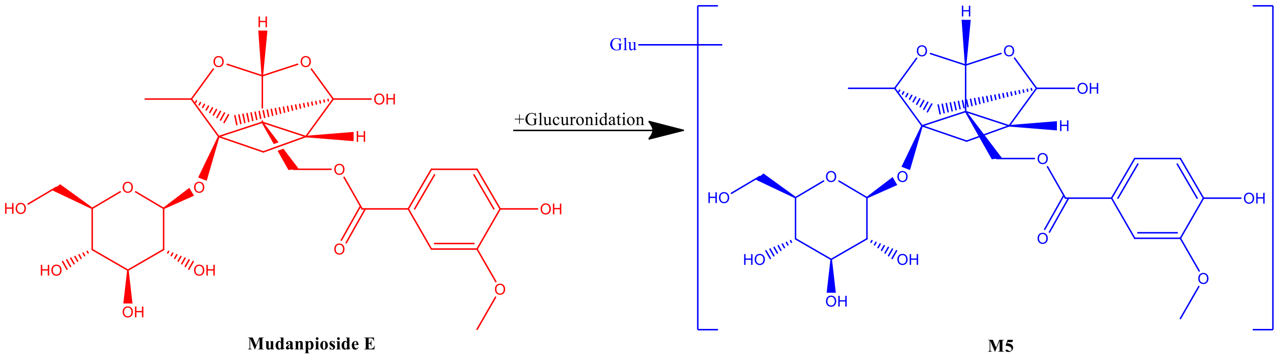

Fig. S12. The proposed metabolic pathways of Mudanpioside E

The compounds could only be detected in mesenteric blood were colored in blue; the compounds could both be detected in mesenteric blood and systemic blood stream were colored in red.
